# Loss of quality of life and increased societal costs in patients with hypertrophic cardiomyopathy: the AFFECT-HCM study

**DOI:** 10.1093/ehjqcco/qcae092

**Published:** 2024-11-08

**Authors:** Stephan A C Schoonvelde, Isabell Wiethoff, Peter-Paul Zwetsloot, Alexander Hirsch, Christian Knackstedt, Tjeerd Germans, Maurits Sikking, Arend F L Schinkel, Marjon A van Slegtenhorst, Judith M A Verhagen, Rudolf A de Boer, Silvia M A A Evers, Mickaël Hiligsmann, Michelle Michels

**Affiliations:** Department of Cardiology, Erasmus MC, Cardiovascular Institute, Thorax Center, Dr. Molewaterplein 40, 3015 GD, Rotterdam, The Netherlands; Department of Health Services Research, Care and Public Health Research Institute (CAPHRI), Maastricht University, 6229 ER, Maastricht, The Netherlands; Department of Cardiology, Erasmus MC, Cardiovascular Institute, Thorax Center, Dr. Molewaterplein 40, 3015 GD, Rotterdam, The Netherlands; Netherlands Heart Institute, 3511 EP, Utrecht, The Netherlands; Department of Cardiology, Erasmus MC, Cardiovascular Institute, Thorax Center, Dr. Molewaterplein 40, 3015 GD, Rotterdam, The Netherlands; Department of Radiology and Nuclear Medicine, Erasmus MC, 3015 GD, Rotterdam, The Netherlands; Department of Cardiology and Cardiovascular Research Institute Maastricht (CARIM), Maastricht University Medical Center+, 6229 HX, Maastricht, The Netherlands; Department of Cardiology, Northwest Clinics, 1815 JD, Alkmaar, the Netherlands; Department of Cardiology and Cardiovascular Research Institute Maastricht (CARIM), Maastricht University Medical Center+, 6229 HX, Maastricht, The Netherlands; Department of Cardiology, Erasmus MC, Cardiovascular Institute, Thorax Center, Dr. Molewaterplein 40, 3015 GD, Rotterdam, The Netherlands; Department of Clinical Genetics, Erasmus MC, 3015 GD, Rotterdam, The Netherlands; Department of Clinical Genetics, Erasmus MC, 3015 GD, Rotterdam, The Netherlands; Department of Cardiology, Erasmus MC, Cardiovascular Institute, Thorax Center, Dr. Molewaterplein 40, 3015 GD, Rotterdam, The Netherlands; Department of Health Services Research, Care and Public Health Research Institute (CAPHRI), Maastricht University, 6229 ER, Maastricht, The Netherlands; Trimbos Institute, Netherlands Institute of Mental Health and Addiction, Centre for Economic Evaluation, 3521 VS, Utrecht, The Netherlands; Department of Health Services Research, Care and Public Health Research Institute (CAPHRI), Maastricht University, 6229 ER, Maastricht, The Netherlands; Department of Cardiology, Erasmus MC, Cardiovascular Institute, Thorax Center, Dr. Molewaterplein 40, 3015 GD, Rotterdam, The Netherlands

**Keywords:** Hypertrophic cardiomyopathy, Quality of life, Healthcare, Societal costs, Economic burden, Burden of disease

## Abstract

**Introduction:**

Hypertrophic cardiomyopathy (HCM) is the most prevalent inherited cardiac disease. The impact of HCM on quality of life (QoL) and societal costs remains poorly understood. This prospective multi-centre burden of disease study estimated QoL and societal costs of genotyped HCM patients and genotype-positive phenotype-negative (G+/P−) subjects.

**Methods and results:**

Participants were categorized into three groups based on genotype and phenotype: (i) G+/P− [left ventricular (LV) wall thickness <13 mm], (ii) non-obstructive HCM [nHCM, LV outflow tract (LVOT) gradient <30 mmHg], and (iii) obstructive HCM (oHCM, LVOT gradient ≥30 mmHg). We assessed QoL with EQ-5D-5L and Kansas City Cardiomyopathy Questionnaires (KCCQ). Societal costs were measured using medical consumption (Medical Consumption Questionnaire) and productivity cost (iMTA Productivity Cost Questionnaire) questionnaires. We performed subanalyses within three age groups: <40, 40–59, and ≥60 years. From three Dutch hospitals, 506 subjects were enrolled (84 G+/P−, 313 nHCM, 109 oHCM; median age 59 years, 39% female). HCM (both nHCM and oHCM) patients reported reduced QoL vs. G+/P− subjects (KCCQ: 88 vs. 98, EQ-5D-5L: 0.88 vs. 0.96; both *P* < 0.001). oHCM patients reported lower KCCQ scores than nHCM patients (83 vs. 89, *P* = 0.036). Societal costs were significantly higher in HCM patients (€19,035/year vs. €7385/year) compared with G+/P− controls, mainly explained by higher healthcare costs and productivity losses. Being symptomatic and of younger age (<60 years) particularly led to decreased QoL and increased costs.

**Conclusion:**

HCM is associated with decreased QoL and increased societal costs, especially in younger and symptomatic patients. oHCM patients were more frequently symptomatic than nHCM patients. This study highlights the substantial disease burden of HCM and can aid in assessing new therapy cost-effectiveness for HCM in the future.

## Graphical Abstract

**Graphical Abstract:**
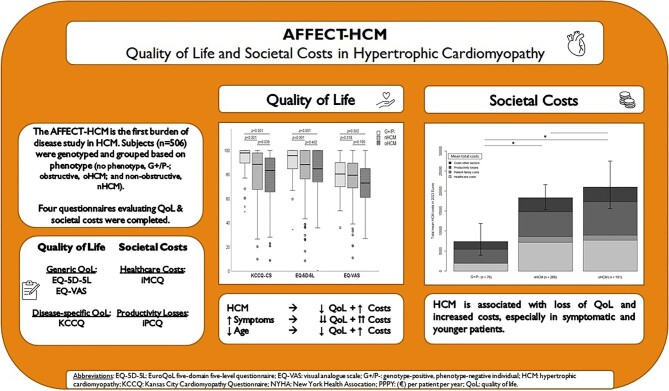

Key Learning Points What is already known

- Hypertrophic cardiomyopathy (HCM) is a prevalent myocardial disease, affecting 1 in 500 individuals and frequently causing limiting cardiac symptoms. Approximately half of these individuals have a genetic predisposition.
- Individuals with a genetic predisposition do not always develop a HCM phenotype (genotype-positive, phenotype-negative; G+/P−), and these individuals undergo regular cardiologic evaluation to assess potential phenotype development.
- Previous studies on the burden of disease in HCM have primarily focused on healthcare costs, often neglecting the broader societal and work-related economic impacts, particularly in G+/P− individuals.

What this study adds

- This burden of disease study provides a comprehensive analysis of both the quality of life and societal costs associated with HCM and G+/P− individuals.
- Symptomatic HCM patients face a marked reduction in quality of life and significant societal costs, which is particularly pronounced in younger patients.
- The study highlights that productivity losses are major contributors to the societal burden of HCM, underscoring the need for targeted therapy strategies to mitigate long-term societal costs.

## Introduction

Hypertrophic cardiomyopathy (HCM) is a myocardial disease characterized by left ventricular (LV) hypertrophy in the absence or in excess of abnormal loading conditions.^1^ It is the most common inherited cardiac disease, affecting 1 in 200–500 people worldwide.^2,3^ Based on the clinical phenotype, HCM patients are divided between obstructive HCM (oHCM) in the presence of a maximal LV outflow tract (LVOT) obstruction ≥30 mmHg, and otherwise non-obstructive HCM (nHCM). Patients with HCM may experience exertional dyspnoea, chest pain, syncope, and both atrial and ventricular arrhythmias.^4,5^ A pathogenic DNA variant is identified in approximately half of HCM cases, most frequently in genes encoding proteins responsible for sarcomere assembly. After identifying a pathogenic DNA variant, pre-symptomatic genetic testing of family members at risk is recommended.^6^ This cascade screening may lead to the identification of relatives with a pathogenic DNA variant, but without an HCM phenotype (genotype-positive, phenotype-negative; G+/P−). Both HCM patients as well as G+/P− relatives may be challenged by physical, social, and psychological burdens, which in combination with external and internal stressors and anxiety can lead to a decreased quality of life (QoL), lower participation in daily activities, and increased healthcare and societal costs.^7^

Burden of disease (BoD) studies measure the impact of different aspects of a health condition on society, which can then be used to inform healthcare policy and decision-making.^8^ Important information is gathered on healthcare, societal, and work-related costs of illness and QoL data, which allows for health and social care optimization from the individualized patient to the healthcare system in general.^9^ To date, few data are available on the societal and economic burden of HCM, especially for G+/P− individuals. Recent cost-of-illness studies have attempted to estimate the economic burden of HCM, but left out societal and work-related costs such as productivity losses, which might be substantial given the relatively young age of HCM patients.^10–13^

The AFFECT-HCM (Qu**a**lity o**f** Li**fe** and **C**os**t**s in **HCM**) study is to our knowledge the first study broadly estimating the QoL and 1-year societal costs of HCM and G+/P− subjects. This study provides generic and disease-specific information on QoL and elucidates the healthcare and societal costs of HCM in a multi-centre setting within the Netherlands. This comprehensive approach enables an assessment of the impact of HCM from a holistic, patient-centred perspective.

## Methodology

The study was conducted according to the principles of the Declaration of Helsinki and was approved by the Institutional Review Board of the Erasmus University Medical Centre Rotterdam (MEC-2022-0036), and subjects provided verbal and written informed consent.

### Study design and population

The design of the AFFECT-HCM study has been published previously.^14^ In short, the AFFECT-HCM is a multi-centre prospective observational cohort BoD study consecutively including both genotyped HCM patients and G+/P− subjects, aged 18–80 years, approached from the outpatient clinics of the Erasmus Medical Centre (Rotterdam, the Netherlands), Maastricht University Medical Centre (Maastricht, the Netherlands), and Northwest Hospital Group (Alkmaar, the Netherlands).

The 2015 American College of Medical Genetics and Genomics/Association for Molecular Pathology guidelines were used to interpret gene variants.^15^ Subjects with a pathogenic/likely pathogenic (P/LP) variant were classified as G+. Phenotype was assessed at baseline with electrocardiogram (ECG) and echocardiography performed ≤6 months from inclusion. HCM was diagnosed according to the guidelines by the presence of a maximal LV wall thickness ≥15 mm, or ≥13 mm in G+ subjects.^16^ Apical HCM was diagnosed according to the diagnostic criteria specified for this phenotype.^17^ The HCM phenotype was further defined by the presence of LVOT obstruction at inclusion (maximal LVOT gradient ≥30 mmHg) as obstructive HCM (oHCM) or nHCM in the absence of LVOT obstruction. References to ‘HCM’ patients encompass both nHCM and oHCM patients. G+ subjects, without a phenotype (maximal LV wall thickness <13 mm), were classified as G+/P−.

### Data collection and study assessments

A medical history was constructed from participant medical dossiers. Cardiac symptoms and New York Heart Association (NYHA) class were assessed on the day of study inclusion. Symptomatic status was defined as any individual in NYHA class II–IV. Vital signs (height, weight, blood pressure, and heart rate) were recorded after ECG assessment. Sport participation was quantified upon inclusion and defined as at least 1 day a week of 30 min of moderate-to-vigorous exercise, or at least 2 days a week of 15 min of moderate-to-vigorous exercise. A composite cardiovascular risk profile was defined by the presence of obesity (body mass index ≥30 kg/m^2^), a history of arterial hypertension, diabetes mellitus, and/or hypercholesterolaemia. Coronary artery disease was defined as: prior myocardial infarction, history of percutaneous coronary intervention, or coronary artery bypass grafting. The HCM SCD Risk score was calculated using data available at the time of inclusion.^18^ Patients with prior septal reduction therapy were excluded from the risk score calculation because of the associated reduction of maximal wall thickness and LVOT gradient, both parameters in the HCM SCD Risk score. Medication use was recorded for any prescription medication intended for regular use. Cardiac medications were defined as any medication in the following groups: anti-arrhythmic drugs, beta-blockers, calcium channel blockers, digoxin, ivabradine, loop diuretics, nitrates, oral anticoagulants, platelet aggregation inhibitors, renin-angiotensin-aldosterone system inhibitors (including angiotensin receptor-neprilysin inhibitors), sodium-glucose cotransporter-2 inhibitors, statins, and ezetimibe.

Questionnaires were provided to the patient via an electronic environment upon study inclusion without investigator input. Generic and disease-specific QoL was measured using two validated questionnaires. The generic EuroQoL five-domain five-level (EQ-5D-5L) questionnaire was used to assess five health dimensions (mobility, self-care, usual activities, pain/discomfort, and anxiety/depression), along with the attached visual analogue scale (EQ-VAS).^19^ The EQ-5D-5L can be translated into a utility score, where 0 represents death and 1 represents perfect health. Scores below 0 are possible, representing a health status ‘worse than death’. Utility scores were obtained from a value reference set of the general Dutch population.^20^ The EQ-VAS is a self-reported scale from 0 to 100 asking the subject to rate their health on that day using a sliding scale. Disease-specific QoL was measured with the Kansas City Cardiomyopathy Questionnaire (KCCQ), covering seven health domains (symptom frequency, symptom burden, symptom stability, physical limitations, social limitations, QoL, and self-efficacy).^21^ All dimensions range from 0 (worst functioning) to 100 (excellent health). The KCCQ has been previously validated in HCM patients.^22,23^ An absolute difference of at least five points is considered clinically important.^21^ Although all subscores from the KCCQ are reported, the KCCQ-Clinical Summary Score is primarily discussed, as it best mirrors the key concepts of the NYHA classification.^21^

Cost data were obtained with two questionnaires from the Institute for Medical Technology Assessment (iMTA): the Medical Consumption Questionnaire (iMCQ) and iMTA Productivity Cost Questionnaire (iPCQ).^24,25^ Medical consumption costs were quantified with the iMCQ, which measures the usage of a wide range of healthcare services including the general practitioner and practitioner assistants, physiotherapy, social worker, occupational therapists, dieticians, homeopathy, psychotherapy, company physician, home care (organization), medicine use, emergency care (emergency room and ambulance), specialist care, hospital care (treatments and overnight stays), and care at other facilities (day and overnight stays). The iMCQ was further amplified by asking subjects to specify the frequency of cardiovascular and non-cardiovascular examinations and interventions they underwent. Medications were individually priced based on frequency and dose per medicine using Dutch retail prices in 2023.^26^ We omitted expensive oncological drugs which were considerably more expensive than the usual drugs for HCM patients in order to minimize data bias and skewing by singular outliers. For patient and family costs, hours of unpaid home care provided by family members and travel expenses for hospital consults were assessed. As these measurements are retrospective and may be subject to potential recall-bias, a recall-period of 3 months is used in these validated questionnaires.^27^ Costs were determined by multiplying the amount/hours of healthcare services by standard prices listed in the Dutch Healthcare Institute costing guidelines.^28^ For costs not listed there, prices were taken from the Dutch Healthcare Authority. These assumptions are elaborated within the supplement (Supplementary material online, *Table S1*).

Productivity losses for paid work and costs in other sectors (e.g. voluntary work, housework, leisure, etc.) were measured with the iPCQ. This questionnaire assesses the individual's ability to work, the amount of lost hours due to illness (absenteeism), the reduced productivity at work due to illness (presenteeism), and the amount of lost hours for unpaid activities (costs in other sectors) over a recall period of 4 weeks. Lost working hours/days were multiplied with standard tariffs as described in the costing guideline.^28^ Productivity losses were calculated with the friction cost approach that is recommended by the Dutch guidelines.^28^ This method values all lost productivity until a worker returns to their duties (short-term absence) or until the worker is replaced (long-term absence or disability). The friction period, i.e. the time needed to replace a sick worker, as calculated with recent Dutch Central Bureau of Statistics data, is 19 weeks for the year 2023.^29^ After this period, the calculation of productivity losses due to long-term absence or disability stops.

Healthcare resource use, and patient and family resource use, were measured using a 3-month recall period. For comparability with other similar studies, the final costs were extrapolated to 1 year, reported as costs per patient per year (PPPY) and reported in 2023 Euro, adjusted for Dutch inflation data as reported by the Dutch Central Bureau of Statistics.^30^ By exception, for G+/P− subjects, costs for cardiovascular examinations were not extrapolated, as these are performed with varying frequency exceeding 1 year per follow-up moment. Productivity losses were measured using a 4-week recall period and extrapolated to the pre-defined friction period, which can be a maximum of 19 weeks. As the time period did not exceed 1 year, no discounting was applied. The supplemental material (Supplementary material online, *Table S1*) summarize all reference prices and assumptions made.

For between-group comparisons, five main pre-defined cost categories were represented.^28^

- *Healthcare costs*: all healthcare expenses directly related to the condition and/or that occur during normal life years.
- *Patient and family costs*: expenses incurred due to travel to a healthcare practitioner, time costs, costs of informal healthcare.
- *Productivity losses*: costs of absenteeism or presenteeism.
- *Other sector costs*: costs incurred in sectors outside of healthcare, e.g. by municipalities, in education, by volunteers (i.e. lost hours/days for unpaid activities), etc.
- *Total societal costs*: a mean summed total cost of all the above costs combined.

### Statistical analysis

A sample size of at least 200 participants was deemed sufficient based on previous BoD studies.^31^ In the interest of performing subgroup analyses, 500 subjects were targeted. Main statistical analyses were conducted using IBM SPSS Statistics version 28 (SPSS, IBM, Armonk, NY, USA). The cost analysis and non-parametric bootstrapping were performed in R version 4.4.0. Baseline characteristics and secondary outcomes were analysed using the chi-square (χ^2^) test for categorical data, the independent-samples *t*-test, Wald test or analysis of variance for normally distributed continuous data, and the Mann–Whitney U or Kruskal–Wallis H test for non-normally distributed continuous and ordinal data. Normality of data was assessed using the Kolmogorov–Smirnov test (as subgroups exceeded *n* ≥50).^32^ Data displaying a normal distribution was reported using the mean with SD, and median with interquartile ranges (IQR) for data not conforming to the normal distribution. Correlation analysis for non-parametric data was performed using the Spearman's rank correlation test. Age-stratified comparisons were conducted in 10- and 20-year intervals to assess age-stratified differences, based on previously determined important HCM age thresholds.^4^ Tests were two-sided and statistical significance was assumed for *P <* 0.05. Given the observational methodology, the same alpha level was maintained during all analyses. Regarding missing data, no imputation or inference was conducted. A complete case analysis was chosen *post hoc* for the questionnaire outcome analysis, considering a response rate of >90% and the absence of significant between-group disparities (except for somewhat younger age) observed between non-respondents and respondents.

As costs showed a high degree of skewness, non-parametric bootstrapping (2000 replications) was performed and corresponding 95% bootstrapped confidence intervals (95% CI) were computed based on the percentile method. Corresponding 95% CIs were compared to determine between-group differences (Bayesian group comparison). According to this method, we can assume with 95% confidence that the difference between the two groups is significant if their bootstrapped 95% CIs do not overlap, and no *P*-value can be provided. For transparency, the non-bootstrapped cost data were represented in the supplement. Despite the non-normal cost data distribution, these costs were represented as mean data as recommended in healthcare costs analysis.^33,34^ As such, supplemental between-group differences were tested using parametric tests (*t*-test or analysis of variance).^34^ To illustrate the data skewness, IQRs were reported next to the supplemental mean cost data.

Several sensitivity and scenario analyses were performed to test the robustness of the findings. First (SA1), societal costs were compared with costs from a healthcare perspective. Second (SA2), similarly to G+/P− subjects, we did not extrapolate cardiovascular examinations for HCM patients and compared the costs of both scenarios. Third (SA3), we calculated the costs for HCM patients when considering all medications and contrasted them to the costs calculated with the cut-off point for expensive oncological medications. Lastly (SA4), the human capital approach was chosen as an alternative method for the calculation of productivity losses. This method does not stop the calculation of productivity losses after 19 weeks, but extrapolates those to the whole year.

## Results

### Participant demographics and phenotype

Participants were recruited between November 2022 and December 2023. In total, 506 subjects were included: 84 G+/P− subjects, 313 nHCM and 109 oHCM patients. Medical baseline data was obtained for all subjects (*Table 1*). Questionnaire completion rate was 93% (*n* = 471) for the EQ-5D-5L and KCCQ, and 92% (*n* = 465) for the iMCQ and iPCQ. Non-responders were younger (median age 51 vs. 59 years, *P* = 0.017), but no other differences were found. The median age of the cohort was 59 (45–67) years and 39% were women. HCM patients were older (60 vs. 45 years, *P* < 0.001), more often male (67%, *P* < 0.001) and had a higher prevalence of cardiovascular risk factors (54% vs. 19%, *P* < 0.001), compared with G+/P− subjects. Including the 84 G+/P− subjects, 312 (62%) subjects carried a P/LP gene variant. Of these, 216 (69%) were *MYBPC3* variants and 50 (16%) were *MYH7* variants. Nine subjects carried two distinct P/LP gene variants, two without HCM. When patients post-septal reduction therapy were included in the oHCM group, oHCM patients were less likely G+ (86, 38% G+ vs. 110, 57% G−; *P* < 0.001). G− HCM patients were older (62 vs. 54 years, *P* < 0.001) and had more cardiovascular risk factors (64% vs. 27%, *P* < 0.001) compared with G + HCM patients. As compared with nHCM, oHCM patients were older (63 vs. 59 years, *P* = 0.010), more symptomatic (55% vs. 35%, *P* < 0.001) and used more cardiac medications (91% vs. 78%, *P* = 0.004), whereas a history of atrial tachyarrhythmias and non-sustained ventricular tachycardias was more common in nHCM patients. Limiting symptoms were reported by 40% (*n* = 169) of HCM patients. Dyspnoea (39%), palpitations (24%), fatigue (23%), and chest pain (18%) were the most commonly reported symptoms. Syncope within 1 year of study inclusion was reported by 11 (3%) subjects. A minority of nHCM patients were in NYHA class II–III (108; 35%, *P* < 0.001). In comparison, most (60; 55%, *P* < 0.001) oHCM patients were in NYHA class II–III. All G+/P− subjects were in NYHA class I.

**Table 1:** Baseline demographics

Characteristics	G+/P− (*n* = 84)	HCM (*n* = 422)	*P* value	nHCM (*n* = 313)	oHCM (*n* = 109)	*P* value
Age (yrs, IQR)	45 (35–56)	60 (49–67)	<0.001	59 (47–67)	63 (57–69)	0.010
Female (*n*, %)	57 (68)	140 (33)	<0.001	101 (32)	39 (36)	0.502
BMI (kg/m^2^, SD)	25.0 (±4.6)	27.5 (±4.2)	<0.001	27.5 (±4.3)	27.6 (±4.0)	0.782
Active employment (*n*, %)	71 (85)	247 (59)	<0.001	185 (59)	62 (57)	0.685
Sport participation (*n*, %)	50 (60)	209 (50)	0.094	163 (52)	46 (42)	0.076
Medical history						
Hypertension (*n*, %)	8 (10)	148 (35)	<0.001	105 (34)	43 (39)	0.266
Diabetes mellitus (*n*, %)	4 (5)	43 (10)	0.118	29 (9)	14 (13)	0.287
Dyslipidaemia (*n*, %)	3 (4)	70 (17)	0.002	48 (15)	22 (20)	0.241
CAD (*n*, %)	0	27 (6)	0.017	20 (6)	7 (6)	0.991
Medication use (*n*, %)	29 (35)	354 (84)	<0.001	253 (81)	101 (93)	0.004
Cardiac medication (n, %)	15 (18)	342 (81)	<0.001	243 (78)	99 (91)	0.002
HCM characteristics						
Age of diagnosis (yrs, IQR)	-	49 (37–60)	-	47 (35–58)	54 (46–63)	<0.001
P/LP gene variant (*n*, %)	84 (100)	228 (54)	<0.001	192 (61)	36 (35)	<0.001
NYHA classification (*n*, %)	-		-			<0.001
NYHA I		253 (60)		205 (65)	49 (45)	<0.001
NYHA II		142 (34)		100 (32)	41 (38)	0.280
NYHA III		27 (6)		8 (3)	19 (17)	<0.001
HCM SCD Risk score (IQR)	-	2.76% (1.53–4.09)	-	2.09% (1.27–3.54)	3.79% (2.83–6.10)	<0.001
HCM-related therapy (*n*, %)						
Beta-blocker	7 (8)	236 (56)	<0.001	170 (54)	66 (61)	0.259
Non-DHP CCB	1 (1)	68 (16)	<0.001	39 (12)	29 (27)	<0.001
SRT (*n*, %)	-	102 (24)	-	87 (28)	15 (14)	0.003
Myectomy		84 (82)		72 (83)	12 (80)	
Alcohol septal ablation		15 (15)		13 (15)	2 (13)	
Both		3 (3)		2 (2)	1 (7)	
Atrial fibrillation (*n*, %)	0	103 (24)	<0.001	89 (28)	14 (13)	<0.001
Paroxysmal		81 (79)		69 (78)	12 (85)	
Permanent		22 (21)		20 (22)	2 (15)	
NSVT (n, %)	1 (1)	126 (30)	<0.001	107 (34)	20 (18)	0.002
ICD (*n*, %)	0	115 (27)	<0.001	93 (30)	22 (20)	0.054
Primary prevention ICD		96 (83)		76 (82)	20 (91)	0.297
Echocardiography						
LA diameter (mm, SD)	35 (±5)	45 (±8)	<0.001	45 (±8)	46 (±7)	0.564
MWT (mm, SD)	9 (±4)	17 (±4)	<0.001	16 (±4)	18 (±4)	<0.001
LVEF <50% (*n*, %)	1 (1)	22 (5)	0.104	22 (7)	0	0.005
LVOT gradient (mmHg, SD)						
At rest	5 (±3)	17 (±26)	<0.001	7 (±4)	46 (±39)	<0.001
After valsalva	5 (±3)	31 (±37)	<0.001	11 (±6)	80 (±37)	<0.001

The HCM SCD Risk score was calculated only in patients without prior SRT.

BMI, body mass index; CAD, coronary artery disease; ICD, implantable cardioverter defibrillator; LA, left atrium; LVEF, left ventricular ejection fraction; LVOT, left ventricular outflow tract; MWT, maximal wall thickness; non-DHP CCB, non-dihydropyridine calcium channel blocker; NSVT, non-sustained ventricular tachycardia; P/LP, pathogenic/likely pathogenic gene variant; SCD, sudden cardiac death; SD, standard deviation; SRT, septal reduction therapy.

### Quality of life

**Figure 1:**
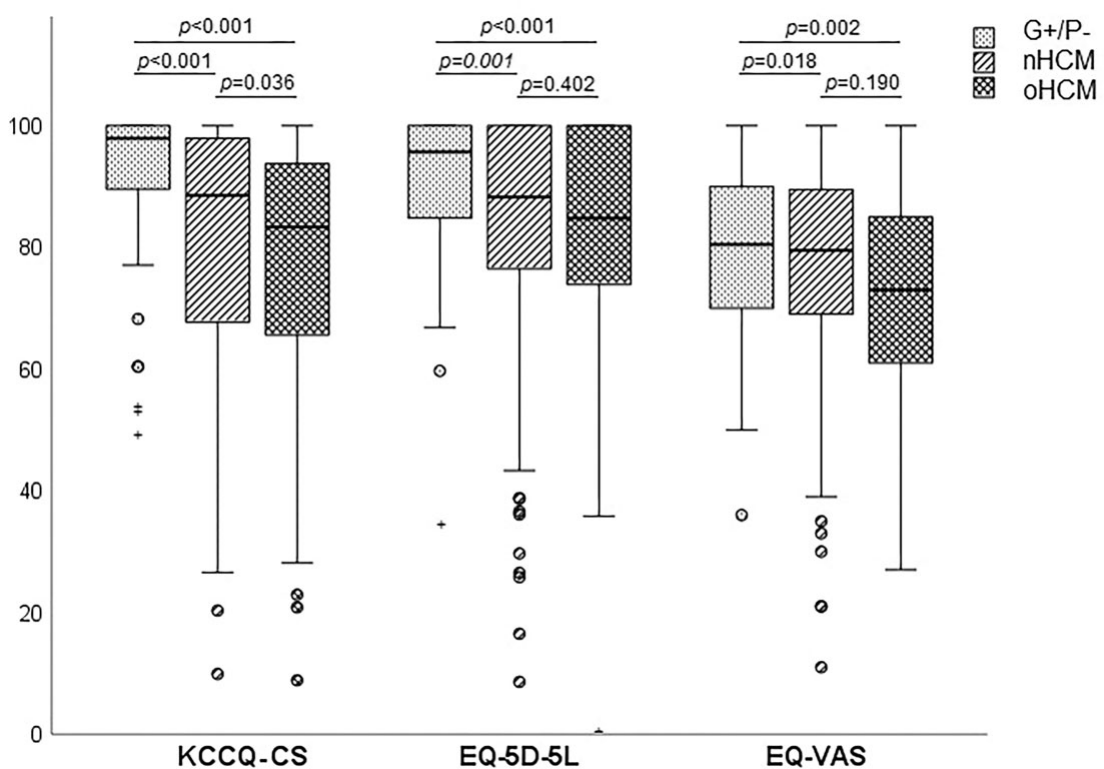
Quality of life questionnaire scores per phenotype represented as box plots. The EQ-5D-5L utility score was multiplied by a factor of 100 to allow for visual comparison.

The QoL questionnaire data displayed a non-normal distribution with a negative skew, even within symptomatic patients. Bootstrapped EQ-5D-5L utilities and EQ-VAS are available in the supplement (Supplementary material online, *Table S2*). As shown in *Figure 1*, G+/P− subjects reported high QoL, with maximal KCCQ scores, high utilities and high EQ-VAS values, the latter comparable with the Dutch average reference set (0.96 vs. 0.87, and 81 vs. 81). HCM patients reported lower QoL on all questionnaires compared with the G+/P− cohort (*Table 2*). For HCM patients, the KCCQ-CS was 88, utilities were 0.88 and the EQ-VAS was 77. oHCM patients had lower KCCQ-CS scores than nHCM patients (83 vs. 89, *P* = 0.036). Higher NYHA classification correlated with decreasing KCCQ-CS, utilities, and EQ-VAS scores (all *P* < 0.001).

**Table 2:** All median quality of life-related questionnaire outcomes of the respondents, with comparisons between phenotypes

	KCCQ-CS	*P* value	EQ-5D-5L	*P* value	EQ-VAS	*P* value
G+/P− (*n* = 78)	98 (90–100)	<0.001	0.96 (0.85–1)	<0.001	81 (70–90)	0.006
All HCM (*n* = 393)	88 (67–98)		0.88 (0.76–1)		77 (66–89)	
Asymptomatic HCM (*n* = 235)	95 (85–100)	<0.001	0.91 (0.82–1)	<0.001	80 (70–90)	<0.001
Symptomatic HCM (*n* = 158)	67 (54–83)		0.78 (0.68–0.89)		70 (60–80)	
Non-obstructive HCM (*n* = 292)	89 (68–98)	0.036	0.88 (0.77–1)	0.402	80 (69–90)	0.190
Obstructive HCM (*n* = 101)	83 (65–94)		0.85 (0.73–1)		73 (61–85)	

**Figure 2:**
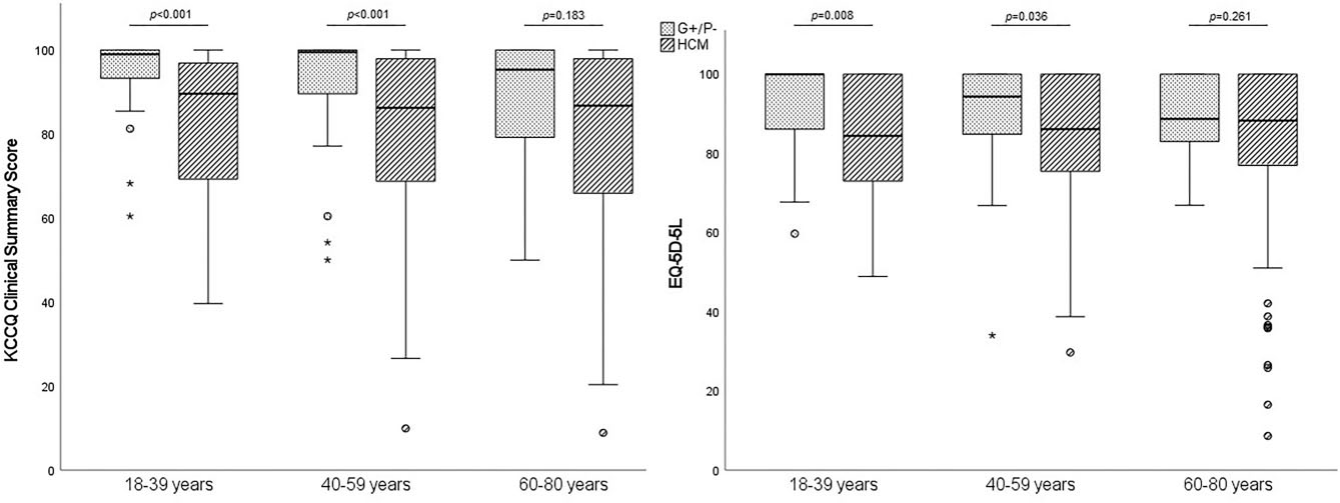
Quality of life as assessed by the Kansas City Cardiomyopathy Questionnaire Clinical Summary Score (KCCQ-CS) and the EuroQoL five-domain 5-level (EQ-5D-5L) questionnaire for both genotype-positive, phenotype-negative (G+/P−) subjects and hypertrophic cardiomyopathy (HCM) patients, stratified by age groups. The EQ-5D-5L utility score was multiplied by a factor of 100 to allow for visual comparison. Data represented as box plots.

Asymptomatic HCM patients reported only marginally reduced KCCQ values but equivalent utilities and EQ-VAS values compared with G+/P− subjects, whereas for both symptomatic and asymptomatic HCM patients, there were no between-group differences in QoL (Supplementary material online, *Table S3*). Female HCM patients reported lower KCCQ-CS scores compared with male HCM patients (83 vs. 89, *P* = 0.027). Younger HCM patients (<60 years) experienced a decreased QoL compared with age-stratified G+/P− peers (*Figure 2*). No important differences were found for age-stratified QoL comparisons between nHCM and oHCM patients (Supplementary material online, *Table S4*). HCM patients engaging in regular exercise reported better QoL (KCCQ-CS: 93 vs, 81, EQ-5D-5L: 0.89 vs. 0.81, EQ-VAS: 80 vs. 71, all *P* < 0.001). Medication use was associated with worse QoL (KCCQ-CS 83 vs. 97, *P* < 0.001; EQ-5D-5L 0.85 vs. 1, *P* < 0.001; EQ-VAS 75 vs. 80, *P* = 0.013). A history of atrial fibrillation (*n* = 103, 24%) correlated with lower QoL (KCCQ-CS 74 vs. 90, *P* < 0.001; EQ-5D-5L 0.82 vs. 0.88, *P* = 0.006; and EQ-VAS 73 vs. 80, *P* = 0.037). Implantable cardioverter-defibrillator (ICD; *n* = 115, 27%) implantation, either for primary or secondary prevention, did not influence QoL (KCCQ-CS 83 vs. 89, *P* = 0.054; EQ-5D-5L 0.88 vs. 0.85, *P* = 0.217; EQ-VAS 80 vs. 75, *P* = 0.389). Excluding G+/P− subjects, G+ status was associated with higher KCCQ-CS scores (89 vs. 83, *P* = 0.007) in HCM patients. There were weak significant correlations [Spearman's ρ = −(0.211–0.149), *P* < 0.05] between the questionnaires and the HCM SCD Risk score (Supplementary material online, *Figure S1*). A full overview of the questionnaires for all the main groups, and for HCM patients divided by NYHA class, can be found in Supplementary material online, *Table S5*.

### Resource use and costs

**Figure 3:**
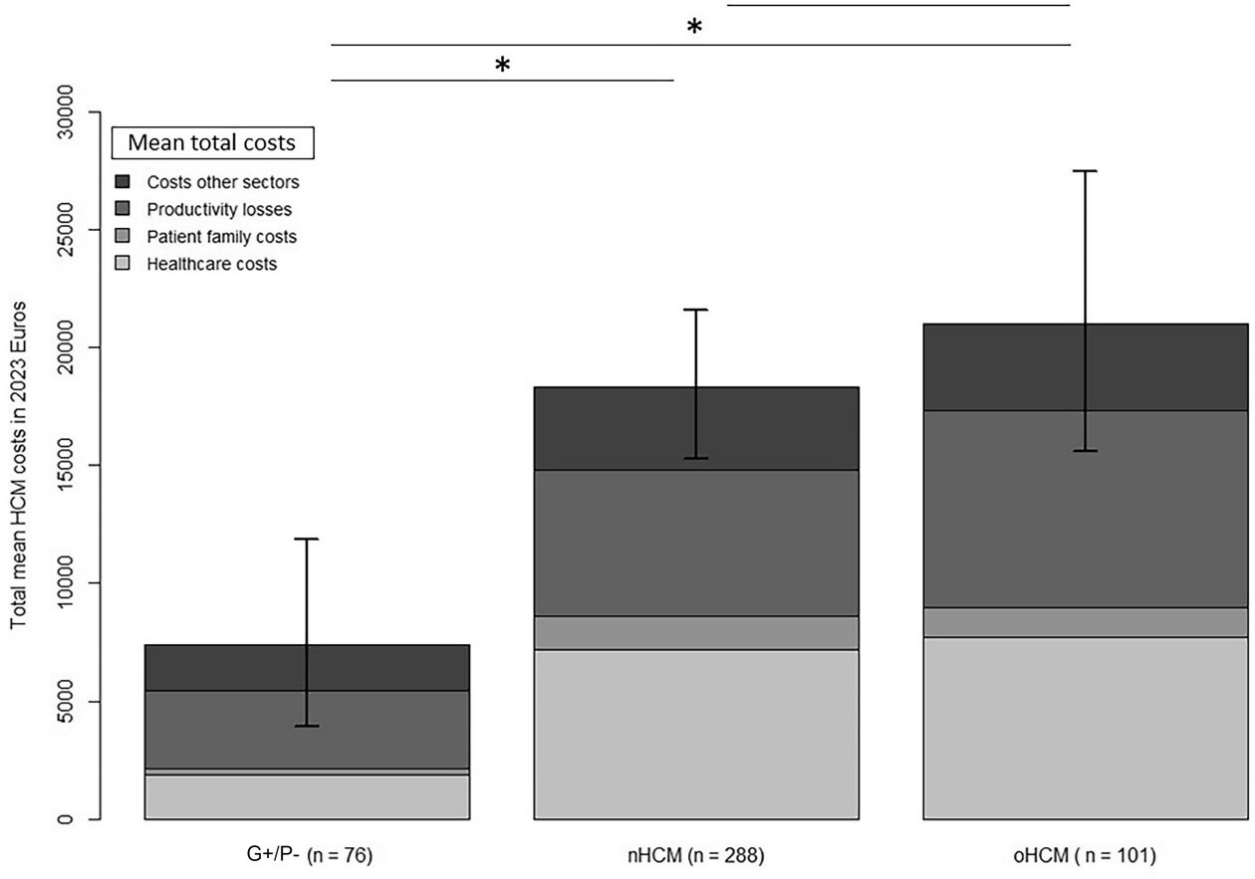
Bootstrapped mean total societal costs (in € per patient per year, PPPY) per patient per phenotype. Bars represent the 95% confidence intervals. Between-group statistical significance is denoted with an *.

Bootstrapped total mean costs were €19,035 PPPY in HCM patients. These were €18,330 PPPY for the nHCM, €21,036 PPPY for the oHCM, and €7385 PPPY G+/P− subgroups. Mean total costs per subgroup are illustrated in *Figure 3*. The detailed overview of every bootstrapped healthcare resource utilization (HCRU) and individual costs for all HCM patients are represented in *Table 3*. A detailed overview of all subgroup costs are available (Supplementary material online, *Tables S6*–*S8*). Overall, HCM patients had considerably increased total societal costs than G+/P− controls (€19,035 vs. €7385 PPPY), explained by higher healthcare costs (€7330 vs. €1872 PPPY), higher productivity losses (€6724 vs. €3282 PPPY), and higher patient and family costs (€1403 vs. €273 PPPY) (*Table 4*). Healthcare costs represented a significant portion of total societal costs, however, the broader non-healthcare costs were, in sum, larger contributors to overall societal expenditures for both G+/P− and HCM subjects (€1872 vs. €5512 and €7330 vs. €11,705, respectively).

**Table 3:** Mean healthcare resource utilization (HCRU) and mean costs per patient per year (PPPY) for hypertrophic cardiomyopathy (HCM) patients according to cost types

HCM (*n* = 389)	HCRU	95% Bootstrapped CI HCRU	Costs PPPY (2023 Euro)	95% Bootstrapped CI Costs
**Healthcare costs**			€7330	[€6334, €8498]
Primary healthcare			€787	[€603, €1022]
General Practitioner	4.0	[3.5, 4.6]	€128	[€111, €147]
Practitioner assistant	1.5	[1.1, 1.9]	€31	[€23, €41]
Social worker	0.4	[0.1, 0.8]	€50	[€12, €104]
Physiotherapy	8.5	[5.1, 13.7]	€344	[€208, €554]
Occupational therapy	0.2	[0, 0.5]	€5	[€0, €12]
Dietitian	0.7	[0.4, 0.9]	€17	[€11, €24]
Homeopathy	0.1	[0.0, 0.3]	€4	[€1, €10]
Psychologist	1.6	[1.0, 2.3]	€165	[€99, €240]
Company physician	1.0	[0.7, 1.3]	€31	[€22, €40]
Homecare (hours)			€304	[€112, €565]
Domestic support	6.7	[2.8, 12.0]	€227	[€96, €409]
Self-care support	1.2	[0, 3.7]	€72	[€0, €221]
Nursing support	0.1	[0.5, 0.9]	€5	[€0, €14]
Emergency care			€298	[€200, €406]
Emergency room	0.7	[0.5, 0.9]	€185	[€124, €248]
Ambulance care	0.2	[0.1, 0.3]	€113	[€68, €175]
Cardiologist care	4.0	[3.6, 4.4]	€501	[€450, €555]
Other specialist care	3.7	[3.0, 4.4]	€463	[€382, €547]
Patient care			€3,581	[€2763, €4525]
Cardiac echo	3.5	[3.2, 3.9]	€373	[€338, €416]
Electrocardiogram	4.5	[4.0, 5.0]	€228	[€204, €254]
MRI	0.6	[0.5, 0.8]	€177	[€131, €222]
Blood testing	3.6	[3.0, 4.3]	€118	[€98, €140]
Ergometry	0.8	[0.7, 1.0]	€127	[€101, €157]
Holter monitoring	1.3	[1.1, 1.5]	€368	[€312, €428]
Hospital procedures	0.9	[0.7, 1.2]	€1,982	[€1562, €2541]
Hospital admissions (admissions per year)	0.4	[0.3, 0.6]	€1,119	[€632, €1753]
Residential home	0	[0, 0]	€0	[€0, €0]
Rehabilitation centre	0.5	[0.1, 1.0]	€451	[€100, €915]
Psychiatric centre	0	[0, 0]	€0	[€0, €0]
Other centres	0.1	[0, 0.2]	€29	[€0, €82]
Cardiac medications			€474	[€403, €545]
Other medications			€921	[€505, €1481]
**Patient family costs**			€1403	[€877, €2056]
Home care	27.4	[14.0, 44.0]	€535	[€274, €858]
Self-care assistance	11.0	[2.8, 23.7]	€215	[€54, €462]
Practical help	26.8	[13.4, 43.9]	€522	[€262, €857]
Hospital travel costs			€131	[€109, €154]
**Productivity losses**			€6724	[€5,365, €8,284]
Short-term sick leave			€1052	[€606, €1578]
Long-term sick leave, no FC			€1399	[€665, €2228]
Long-term sick leave			€1764	[€948, €2648]
Presenteeism			€1521	[€919, €2278]
Disability costs			€989	[€627, €1380]
**Other sector costs**			€3578	[€2410, €4899]
**Mean total costs**			€19,035	[€16,299, €21,974]

A total of 2000 replications were performed.

FC, friction costing; MRI, magnetic resonance imaging.

**Table 4:** Bootstrapped cost data for the different HCM phenotypes and between symptomatic (NYHA II–III) and asymptomatic (NYHA I) HCM patients

Mean costs Bootstrapped € PPPY (95% CI)	G+/P− (*n* = 76)	HCM (*n* = 389)	S	NYHA I HCM (*n* = 233)	NYHA II–III HCM (*n* = 156)	S	nHCM (*n* = 288)	oHCM (*n* = 101)	S
Healthcare costs	**€1872** [€1147, €2732]	**€7330** [€6234, €8498]	*	**€6361** [€5049, €7780]	**€8710** [6935, €10,738]		**€7173** [€5920–€8611]	**€7723** [€5770, €10,293]	
Patient family costs	**€273** [€76, €561]	**€1403** [€877, €2056]	*	**€834** [€404, €1326]	**€2258** [€1093, €3592]		**€1453** [€787, €2239]	**€1262** [€580, €2065]	
Productivity losses	**€3282** [€1382, €5538]	**€6724** [€5365, €8284]		**€5157** [€3612; €6778]	**€7748** [€5447, €10,278]		**€6187** [€4793, €7801]	**€8352** [€5018, €12,507]	
Other sector costs	**€1957** [€187, €4754]	**€3578** [€2410, €4899]		**€1659** [€777, €2758]	**€6426** [€3852, €9213]	*	**€3518** [€2231, €5106]	**€3698** [€1454, €6400]	
Mean total costs	**€7385** [€3913, €11,870]	**€19,035** [€16,299, €21,974]	*	**€14,012** [€10,995, €17,225]	**€25,142** [€20,282, €30,006]	*	**€18,330** [€15,286, €21,593]	**€21,036** [€15,602, €27,474]	

Horizontal between-group statistically significant (S) difference is denoted with an *. There were no statistically significant differences between symptomatic NYHA class II and NYHA class III HCM patients. CI, confidence interval; HCM, hypertrophic cardiomyopathy; nHCM, non-obstructive HCM; NYHA, new york health association, oHCM, obstructive HCM; PPPY: per patient per year.

**Figure 4:**
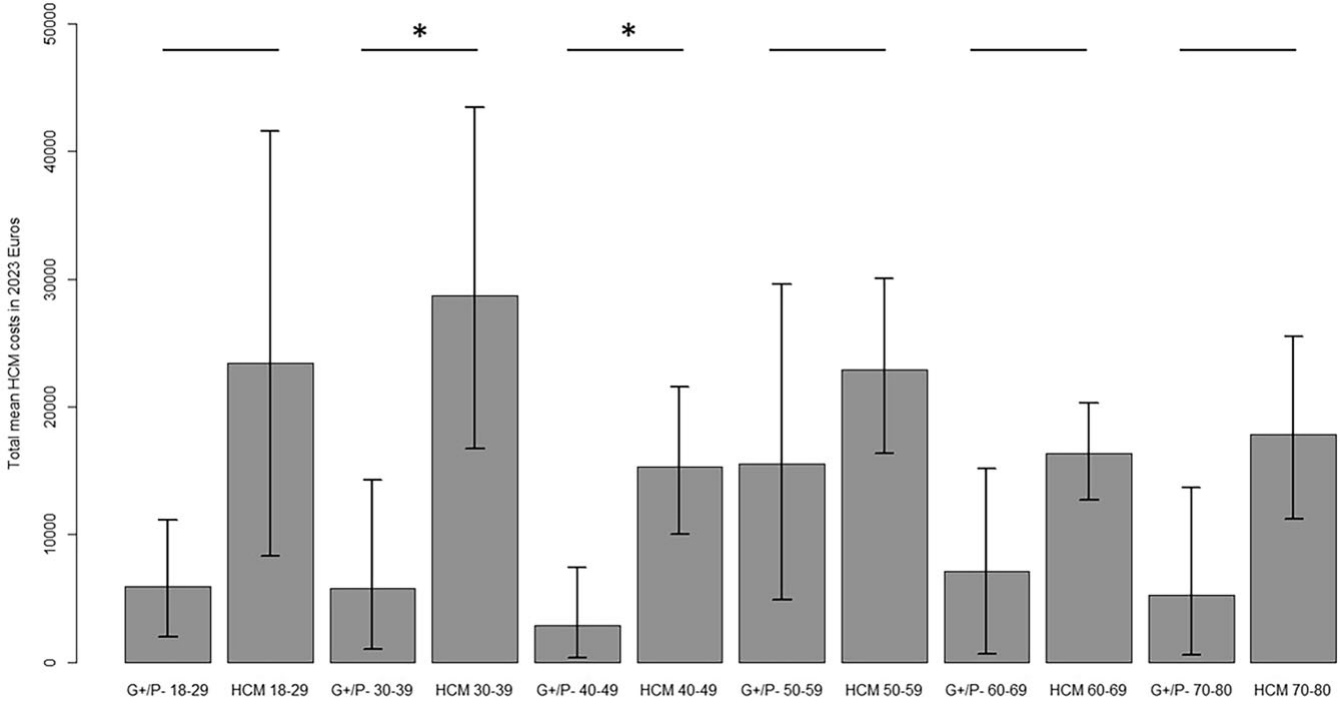
Mean total societal costs (in € per patient per year, PPPY) of both genotype-positive, phenotype-negative (G+/P−) subjects, and hypertrophic cardiomyopathy (HCM) patients, stratified by 10-year age groups. Bars represent 95% confidence intervals. Between-group statistical significance is denoted with an *.

For HCM patients, the biggest cost burden were healthcare costs (€7330 PPPY), directly followed by productivity losses (€6724 PPPY). Productivity losses were considerable, mainly caused by absenteeism (sick leave, €4215 PPPY). Patient and family costs (€1403 PPPY) were mainly driven by the provision of informal home care (e.g. cleaning, groceries) and practical help assistance resources (€535 and €522 PPPY, respectively). Symptomatic HCM patients had considerably higher total costs than asymptomatic patients (€25,142 vs. €14,012 PPPY) (*Table 4*). There were no significant differences in costs between nHCM and oHCM patients, even after excluding asymptomatic patients. Bootstrapped costs for the different NYHA classifications in HCM patients are provided in the supplement (Supplementary material online, *Table S9*). Non-bootstrapped mean costs are also presented in the supplement (Supplementary material online, *Table S10*). Age-stratified mean total yearly costs were relatively the highest in younger (particularly those aged 30–49 years) HCM patients (30–39: €28,691 vs. €5792 and 40–49: €15278 vs. €2881 PPPY), as is illustrated in *Figure 4*. These differences were mainly driven by higher healthcare costs and productivity losses (Supplementary material online, *Figure S2*). Costs were not significantly higher for older (50–80 years) patients than age-stratified G+/P− subjects.

The sensitivity and scenario analyses showed that for both G+/P− subjects and HCM patients, societal costs were significantly higher than costs considered solely from a healthcare perspective. In HCM patients, productivity losses calculated with the human capital approach were significantly higher compared with the friction cost approach. Further analyses (disregarding extrapolation of cardiovascular examinations, and not excluding expensive medications) did not lead to significant differences from the main results (Supplementary material online, *Table S11* and *Figures S3* and *S4*).

## Discussion

The AFFECT-HCM is to our knowledge the first study systematically evaluating the BoD of HCM patients and G+/P− subjects, including broader societal costs. We demonstrate that more than half of HCM patients are asymptomatic, rating their QoL similarly to G+/P− subjects, comparable to the general population. However, symptomatic HCM patients experience a marked reduction in QoL, especially in symptomatic oHCM patients. In particular, younger HCM patients had the most pronounced decrease in QoL and increase in costs based on age-stratified comparisons, particularly due to higher healthcare costs and productivity losses. Total yearly societal costs were considerably higher (+€11,650) for HCM patients in comparison with the G+/P− cohort, mainly driven by higher healthcare costs (+€5458) and productivity losses (+€3442). Symptomatic HCM patients had notably higher total societal costs (+€11,130) than asymptomatic patients. The HCM SCD Risk score demonstrated a weak correlation with the QoL questionnaire outcomes. The HCM SCD Risk score takes into account factors such as left atrial diameter, LVOT gradient, and lower age which relate with phenotype severity, likely explaining this finding. With regards to G+/P− subjects, they showed comparable QoL and costs in comparison to the reference general population. Although the KCCQ was not designed to evaluate healthy individuals, with regard to the EQ-5D-5L, G+/P− subjects showed higher-than-average values compared with the average Dutch population.^19^ Furthermore, their healthcare costs are substantially lower than those in HCM patients. Their primary care HCRU was similar, but inpatient and outpatient hospital care was lower. Productivity losses were present in G+/P− subjects, but lower than the Dutch average (which is approximately €3800 per person in 2023).^35^

Earlier research studying the BoD of HCM has shown similar findings. Christiaans *et al.* previously reported that HCM patients had substantially decreased QoL and increased psychological distress, whereas G+/P− individuals had higher QoL compared with the general population.^36^ Comparing HCM to other cardiomyopathies presents challenges due to varying methodological approaches across studies. Nonetheless, a comparison with the findings from a recent dilated cardiomyopathy BoD study by Wiethoff *et al.* reveals identical EQ-5D-5L outcomes, and furthermore suggests that societal costs are possibly higher for HCM patients (€19,035 vs. €15,407, after adjusting for inflation).^37^ The SHaRe registry reinforces the finding from this study that more severe disease burden is found in younger individuals, which could explain the loss of QoL that particularly younger HCM patients reported in this study.^4^ In the AFFECT-HCM, female patients reported lower KCCQ-CS scores, corresponding with earlier data indicating that female HCM patients tend to be more symptomatic.^38^ This finding is in line with previous studies on general symptom reporting between women and men.^39,40^ Our data reinforces the earlier observation that HCM patients with an ICD do not experience a substantially decreased QoL compared to HCM patients without one.^41^ With regard to the utilized questionnaires, the QoL scores in the AFFECT-HCM are comparable with values reported in literature for other HCM patients using the KCCQ,^22,42,43^ and also similar to the EQ-5D-5L scores for symptomatic oHCM patients in the baseline characteristics of the EXPLORER-HCM trial.^44^

Previous research has often limited its focus exclusively to the healthcare costs associated with HCM, which do not fully capture the broader societal impact. This study introduces novel findings that offer a comprehensive overview of these costs, particularly highlighting the substantial productivity losses due to absenteeism observed in symptomatic and younger patients. This observation is important as it reveals the extensive economic burden that extends beyond direct medical expenses. Given the chronic and progressive nature of HCM, these findings emphasize the need for early and comprehensive management strategies to mitigate long-term societal costs. Furthermore, the structural framework developed from this study provides a valuable benchmark for evaluating the cost-effectiveness of new treatments in the context of upcoming therapies for cardiovascular diseases and cardiomyopathies.

In light of the growing importance of precision medicine, it is necessary to understand the full scope of disease-related societal costs.^45^ This study underscores the potential of novel therapies such as cardiac myosin inhibitors and future gene therapies, to significantly reduce the BoD by improving symptomatology and possibly preventing disease progression.^43,46,47^ Such therapies are particularly promising for younger working-age patients, who were shown to face the most significant societal costs, given that improvements in QoL could lead to lifetime improvements in BoD and therefore decreased lifetime societal costs. The efficacy of cardiac myosin inhibitors in improving QoL and reducing symptoms for oHCM patients has been demonstrated in clinical trials, and is currently under further evaluation in symptomatic nHCM patients.^48,49^ This progression towards novel treatments will reinforce a pivotal role of cost-effectiveness analyses in comprehensively evaluating medical therapies that could alter the disease course of HCM.

There are limitations to this study that need to be addressed. This study utilized patient-reported outcomes, which rely on information provided by participants and may include subjective responses based on estimations. However, previous research has demonstrated that self-reported HCRU yields valid reporting quality.^50^ As there are no reference costs from a general population using this study design, the AFFECT-HCM study utilized the G+/P− subjects as a comparison group, who were younger and had a reversed gender distribution. Although no previous data compared G+/P− subjects to an average population, this subgroup generally had few non-HCM-related comorbidities, making it a reasonable healthy surrogate control group. For the age-stratified comparisons, the age-divided subgroups were relatively small. As the findings with age-stratified HCM patients were markedly different, future research should focus on better understanding the QoL and cost differences within specific HCM age groups, particularly given the prior-mentioned varying disease severity that is dependent on the age of HCM onset.^4^ Although no significant differences were found between symptomatic HCM patients in NYHA class II vs. NYHA class III, the overall sample size of NYHA class III patients was small. Another potential limitation of this study was the methodological extrapolation from 3-month data to 1-year data, which is the norm for BoD studies.^26^ Some patient-specific costs may have had overvalued HCRU values due to this. This is noticeable for cardiologist care HCRU, and particularly ECG and TTE examination HCRU, as by definition, subjects had to have had these investigations for inclusion within the AFFECT-HCM study. In order to allow for comparison with other BoD studies, we did not deviate from this approach. Nonetheless, sensitivity analyses showed that either approach did not significantly alter the values for HCM patients. G+/P− cardiovascular costs were not extrapolated, as described in the methodology.

In conclusion, this study revealed that symptomatic HCM is associated with a high BoD, reduced QoL, and substantial societal costs, primarily driven by increased healthcare expenditures and diminished productivity. Working-age patients experience the most significant decline in QoL and face the highest associated costs and societal burdens. The findings from the AFFECT-HCM and similar future research could help policymakers and insurers shape decisions for future HCM therapies, emphasizing the need for cost-effective, therapeutic, and holistic interventions to improve patient outcomes and reduce the economic burden. Early diagnosis, comprehensive management, and novel precision therapies may improve symptoms, reduce disease progression, and lower societal costs. Additionally, offering holistic interventions may reduce BoD for symptomatic and working-age HCM patients. Future studies should explore the main contributors to the increased BoD in HCM patients, aiding clinicians and healthcare practitioners in targeting interventions that enhance QoL and promote broader societal and labour participation.

## Supplementary Material

qcae092_Supplemental_Files

## Data Availability

The data that support the findings of this study are available from the corresponding author upon reasonable request.
