# Supplementary material for: Loss of quality of life and increased societal costs in patients with hypertrophic cardiomyopathy: the AFFECT-HCM study
Source: Eur Heart J Qual Care Clin Outcomes. 2024 Nov 8;11(2):174–85. doi: 10.1093/ehjqcco/qcae092 (PMC11879321; doi:10.1093/ehjqcco/qcae092)
Supplement: qcae092_Supplemental_Files [file qcae092_supplemental_files.zip › AFFECT-HCM Supplemental Data.docx]

**Supplemental Material**

**Supplemental Table S1** – Overview of reference prices and assumptions made in cost analysis.
CBS data used to transfer 2022 Euros into 2023 Euros: inflation factor = 1.038

| (1) Healthcare costs (iMCQ) | | Unit | 2023 costs | Source |
| --- | --- | --- | --- | --- |
| Outpatient care | General practitioner | Consult | € 32.04 | Dutch Costing guideline (2024) |
|  | Practice assistant (POH) | Consult | € 21.64 | Dutch Costing guideline (2024) |
|  | Social worker | Consult | € 131.83 | Dutch Costing guideline (2024) |
|  | Physiotherapist | Consult | € 40.37 | Dutch Costing guideline (2024) |
|  | Occupational therapist | Consult | € 25.24 | Dutch Costing guideline (2024) |
|  | Speech therapist | Consult | € 42.49 | Dutch Costing guideline (2024) |
|  | Dietician | Consult | € 25.64 | Dutch Costing guideline (2024) |
|  | Homeopath or acupuncturist | Consult | € 32.04 | Assumption based on guideline |
|  | Psychologist | Consult | € 102.36 | Dutch Costing guideline (2024) |
|  | Company physician | Consult | € 32.04 | Assumption based on guideline |
| Homecare | Domestic support | Hours | € 34.00 | Dutch Costing guideline (2024) |
|  | Self-care support | Hours | € 59.77 | Dutch Costing guideline (2024) |
|  | Nursing (per hour) | Hours | € 77.85 | Dutch Costing guideline (2024) |
| Emergency care | First aid (*EHBO*)/ER visit | Visit | € 267.80 | Dutch Costing guideline (2024) |
|  | Ambulance | Ride | € 548.06 | Dutch Costing guideline (2024) |
| Specialist care | Specialist care (average) | Consult | € 124.56 | Dutch Costing guideline (2024) |
| Medications | Cardiac & non-cardiac medications | Defined daily dosage | Individual per drug and dose | Farmacotherapeutisch Kompas  <https://www.farmacotherapeutischkompas.nl> |
| Inpatient care | Examinations at hospital:  - Echo (imaging)  - ECG  - MRI  - Blood test  - Exercise (ergometry) test  - 48h Holter monitoring | Amount  Amount  Amount  Amount  Amount  Amount | € 106.91  € 50.57  € 277.15  € 32.41  € 154.73  € 281.14 | Dutch Costing guideline (2024)  NZa tariff  Dutch Costing guideline (2024)  NZa tariff/Guidelines (2024)  NZa tariff  Assumption based on NZa |
|  | Other diagnostics/treatments at hospital:  *Cardiovascular related:*  - ICD follow-up  - ICD change  - Coronary angiography  - Genetic test  - 24h blood pressure test  *Other:*  - CT scan  - PET scan  - X-ray  - Gastroscopy  - Colonoscopy  - Audiometry (ear test)  - OCT (eye) scan  - Spirometry (lung function)  - Stroboscopy (vocal cord test)  - Allergy check  - Dermatologist check-up | Amount  Amount  Amount  Amount  Amount  Amount  Amount  Amount  Amount  Amount  Amount  Amount  Amount  Amount  Amount  Amount | € 151.77  € 364.44  € 700.00  € 173.64  € 100.55  € 195.14  € 999.59  € 85.29  € 457.77  € 206.12  € 43.16  € 90.00  € 64.05  € 70.00  € 195.92  € 124.56 | NZa tariff  NZa tariff  Assumption based on guideline  NZa tariff  Dutch Costing guideline (2024)  Dutch Costing guideline (2024)  Dutch Costing guideline (2024)  Dutch Costing guideline (2024)  NZa tariff  NZa tariff  NZa tariff  Assumption based on guideline  NZa tariff  Assumption based on guideline  NZa tariff  Dutch Costing guideline (2024) |
|  | Other diagnostics/treatments at different care facility:  - Residential care facility  - Rehabilitation care facility  - Psychiatric facility  - Rehabilitation at hospital | Day stay  Day stay  Day stay  Day stay | € 301.02  € 881.26  € 339.43  € 347.73 | Costs per day:  Dutch Costing guideline (2024)  Dutch Costing guideline (2024)  Dutch Costing guideline (2024)  Dutch Costing guideline (2024) |
|  | Hospitalization incl. staff costs (average) | Overnight stay | € 668.47 | Dutch Costing guideline (2024) |
|  | Overnight stay different facility | Overnight stay | n.a. | - |
| (2) Patient and family costs (iMCQ) | | **Unit** | **2023 costs** | **Source** |
| Homecare done by family member | Domestic support | Hours | € 19.51 | Dutch Costing guideline (2024) |
|  | Self-care support | Hours | € 19.51 | Dutch Costing guideline (2024) |
|  | Practical support | Hours | € 19.51 | Dutch Costing guideline (2024) |
| Travel costs to policlinic | Travelling not applicable | Per km | € 0 | Assumption based on guideline |
|  | Any way (e.g. foot, bike, car, public transport, taxi etc.) | Per km | € 0.27 | Dutch Costing guideline (2024) |
| (3) Productivity losses (iPCQ) | | **Unit** | **2023 costs** | **Source** |
| Productivity losses | Short-term productivity losses | Hours | € 41.40 | Dutch Costing guideline (2024) |
|  | Long-term productivity losses | Hours | € 41.40 | Dutch Costing guideline (2024) |
|  | Friction cost period | Weeks | 19 | Dutch Costing guideline (2024) & CBS data |
|  | Reduced productivity at work | Hours | € 41.40 | Dutch Costing guideline (2024) |
| (4) Costs in other sectors (iPCQ) | |  |  |  |
| Costs in other sectors | Unpaid work losses | Hours | € 19.51 | Dutch Costing guideline (2024) |

**Abbreviations:** iMCQ = medical consumption questionnaire; iPCQ = productivity cost questionnaire; NZa = *Nederlandse Zorgautoriteit* [Dutch Healthcare Authority]; EHBO = *Eerste hulp bij ongevallen* [First aid in case of accidents]; ER = Emergency room; CBS = *Centraal Bureau voor de Statistiek* [Central Bureau for Statistics]; ECG = Electrocardiography; MRI = Magnetic resonance imaging; CT = Computed tomography; PET = Positron emission tomography; OCT= Optical Coherence Tomography

**References:**

Dutch Costing guideline 2024: <https://www.zorginstituutnederland.nl/publicaties/publicatie/2024/01/16/richtlijn-voor-het-uitvoeren-van-economische-evaluaties-in-de-gezondheidszorg>

CBS data inflation adjustment: <https://opendata.cbs.nl/statline/#/CBS/nl/dataset/83131ned/table?fromstatweb>

CBS data friction period calculation: <https://www.cbs.nl/nl-nl/cijfers/detail/84545NED>

**Supplemental Table S2** – Bootstrapped quality of life questionnaire outcomes and corresponding 95% CIs. Significant difference (i.e. 95% CI are not overlapping) is marked with the * notation. Abbreviations: CI: confidence interval; EQ-5D-5L: 5-domain 5-level EuroQoL questionnaire; EQ-VAS: visual analogue score; G+/P-: genotype-positive, phenotype-negative subject; HCM: hypertrophic cardiomyopathy; nHCM: non-obstructive HCM; oHCM: obstructive HCM; NYHA: New York Health Association class; S: statistical significance (horizontal between-group difference is denoted with an *).

| **Quality of Life** | **G+/P-**  (n=78)  Bootstrapped mean [95% CI] | **HCM**  (n=393)  Bootstrapped mean [95% CI] | S | **nHCM**  (n=292)  Bootstrapped mean [95% CI] | **oHCM**  (n=101)  Bootstrapped mean [95% CI] | S |
| --- | --- | --- | --- | --- | --- | --- |
| EQ-5D-5L | 0.90 [0.87; 0.93] | 0.84 [0.82, 0.85] | * | 0.84 [0.82, 0.86] | 0.83 [0.79, 0.86] |  |
| EQ-VAS | 80 [78, 83] | 75 [73, 76] | * | 76 [74, 77] | 73 [70, 76] |  |
|  |  |  |  |  |  |  |
| **Quality of Life** | **HCM NYHA I**  (n=235)  Bootstrapped mean [95% CI] | **HCM NYHA II**  (n=134)  Bootstrapped mean [95% CI] | S | **HCM NYHA II**  (n=134)  Bootstrapped mean [95% CI] | **HCM NYHA III**  (n=24)  Bootstrapped mean [95% CI] | S |
| EQ-5D-5L | 0.89 [0.88, 0.91] | 0.77 [0.74, 0.80] | * | 0.77 [0.74, 0.80] | 0.66 [0.56, 0.75] |  |
| EQ-VAS | 79 [77, 81] | 70 [67, 72] | * | 70 [67, 72] | 62 [56, 69] |  |

**Supplemental Table S3** – Questionnaire outcomes of G+/P- subjects versus asymptomatic nHCM and versus oHCM patients, and between asymptomatic and symptomatic (NYHA II-III) nHCM and oHCM patients. Abbreviations: EQ-5D-5L: five-domain five-level EuroQoL questionnaire; EQ-VAS: EuroQoL visual analogue scale; G+/P-: genotype-positive, phenotype-negative; HCM: hypertrophic cardiomyopathy; KCCQ-CS: Kansas City Cardiomyopathy Questionnaire Clinical Summary Score; nHCM: non-obstructive HCM; NYHA: New York Health Association; oHCM: obstructive HCM.

| **Quality of Life** (IQR) |  |  | **P value** |  | **P value** |
| --- | --- | --- | --- | --- | --- |
|  | **G+/P-**  (n=75) | **Asymptomatic nHCM**  (n = 185) |  | **Asymptomatic oHCM**  (n = 46) |  |
| KCCQ-CS | 98 (90-100) | 95 (86-100) | 0.046 | 94 (83-99) | 0.035 |
| EQ-5D-5L | 0.96 (0.85-1) | 0.91 (0.82-1) | 0.467 | 0.89 (0.81-1) | 0.444 |
| EQ-VAS | 81 (70-90) | 80 (70-90) | 0.700 | 80 (70-90) | 0.285 |
|  |  | |  | |  |
|  | **Asymptomatic nHCM**  (n = 185) | | **Asymptomatic oHCM**  (n = 46) | |  |
| KCCQ-CS | 95 (86-100) | | 94 (83-99) | | 0.461 |
| EQ-5D-5L | 0.91 (0.82-1) | | 0.89 (0.81-1) | | 0.721 |
| EQ-VAS | 80 (70-90) | | 80 (70-90) | | 0.395 |
|  | **Symptomatic nHCM**  (n = 103) | | **Symptomatic oHCM**  (n = 55) | |  |
| KCCQ-CS | 66 (51-81) | | 70 (55-85) | | 0.329 |
| EQ-5D-5L | 0.78 (0.67-0.88) | | 0.78 (0.70-0.91) | | 0.271 |
| EQ-VAS | 70 (60-80) | | 70 (60-81) | | 0.649 |

**Supplemental Table S4** – Questionnaire outcomes of each phenotype stratified by age. Amounts per group “n = a/b” where ‘a’ represents amount of people having completed the questionnaires in the group, and ‘b’ represents the entire group amount. Abbreviations: EQ-5D-5L: five-domain five-level EuroQoL questionnaire; G+/P-: genotype-positive, phenotype-negative; HCM: hypertrophic cardiomyopathy; KCCQ-CS: Kansas City Cardiomyopathy Questionnaire Clinical Summary Score; nHCM: non-obstructive HCM; oHCM: obstructive HCM; EQ-VAS: EuroQoL visual analogue scale.

| **Quality of Life** (IQR) | **G+/P-** | **HCM** | P value | **nHCM** | **oHCM** | P value |
| --- | --- | --- | --- | --- | --- | --- |
| **18-29 yrs** | **n = 12/14** | **n = 14/19** |  | **n = 14** | **n = 0** |  |
| KCCQ-CS | 98 (95-100) | 96 (79-98) | 0.101 | 96 (79-98) | - | - |
| EQ-5D-5L | 0.94 (0.79-1) | 0.87 (0.74-1) | 0.432 | 0.87 (0.74-1) | - | - |
| EQ-VAS | 75 (70-90) | 76 (66-86) | 0.432 | 76 (66-86) | - | - |
| **30-39 yrs** | **n = 18/19** | **n = 31/32** |  | **n = 25** | **n = 6** |  |
| KCCQ-CS | 100 (85-100) | 89 (61-96) | 0.011 | 89 (67-96) | 72 (50-100) | 0.789 |
| EQ-5D-5L | 1 (0.88-1) | 0.82 (0.70-1) | 0.011 | 0.82 (0.67-1) | 0.88 (0.52-1) | 0.865 |
| EQ-VAS | 82 (74-85) | 70 (60-80) | 0.003 | 70 (63-80) | 63 (37-81) | 0.314 |
| **40-49 yrs** | **n = 15/15** | **n = 55/60** |  | **n = 44** | **n = 11** |  |
| KCCQ-CS | 100 (94-100) | 90 (71-98) | 0.003 | 91 (70-99) | 85 (74-90) | 0.463 |
| EQ-5D-5L | 1 (0.88-1) | 0.88 (0.77-1) | 0.007 | 0.87 (0.76-1) | 0.88 (.79-.91) | 0.591 |
| EQ-VAS | 86 (75-95) | 80 (70-90) | 0.074 | 78 (65-90) | 80 (71-90) | 0.519 |
| **50-59 yrs** | **n = 19/22** | **n = 81/85** |  | **n = 63** | **n = 18** |  |
| KCCQ-CS | 97 (86-100) | 85 (66-95) | 0.008 | 85 (64-98) | 85 (73-93) | 0.882 |
| EQ-5D-5L | 0.89 (0.72-1) | 0.85 (0.75-1) | 0.709 | 0.88 (0.76-1) | 0.79 (.75-.92) | 0.435 |
| EQ-VAS | 75 (70-89) | 74 (60-85) | 0.243 | 75 (60-85) | 72 (60-90) | 0.855 |
| **60-69 yrs** | **n = 8/8** | **n = 138/149** |  | **n = 96** | **n = 42** |  |
| KCCQ-CS | 93 (79-99) | 88 (67-98) | 0.492 | 91 (68-100) | 82 (65-96) | 0.080 |
| EQ-5D-5L | 0.89 (0.81-1) | 0.88 (0.77-1) | 0.457 | 0.89 (0.78-1) | 0.82 (.70-.94) | 0.081 |
| EQ-VAS | 88 (73-90) | 80 (67-89) | 0.201 | 80 (70-90) | 70 (60-80) | 0.008 |
| **70-80 yrs** | **n = 6/6** | **n = 74/77** |  | **n = 50** | **n = 24** |  |
| KCCQ-CS | 95 (74-100) | 84 (63-95) | 0.173 | 88 (63-96) | 83 (62-93) | 0.540 |
| EQ-5D-5L | 0.94 (0.79-1) | 0.88 (0.76-1) | 0.433 | 0.87 (0.76-1) | 0.92 (0.77-1) | 0.251 |
| EQ-VAS | 80 (56-98) | 80 (70-90) | 0.920 | 80 (70-90) | 80 (70-90) | 0.894 |

**Supplemental Table S5** – All median quality of life questionnaire outcomes of the respondents. Abbreviations: CS: Clinical Summary Score; EQ-5D-5L: 5-domain 5-level EuroQoL questionnaire; EQ-VAS: visual analogue score; G+/P-: genotype-positive, phenotype-negative subject; HCM: hypertrophic cardiomyopathy; IQR: interquartile range; KCCQ: Kansas City Cardiomyopathy Questionnaire; nHCM: non-obstructive HCM; NYHA: New York Health Association class; oHCM: obstructive HCM; OS: Overall Summary Score; QoL: Quality of Life Score; SB: Symptom Burden Score; SE: Self-Efficacy Score; SL: Social Limitation Score; TS: Total Symptom Score.

| **Quality of Life** (IQR) | **G+/P-**  (n=78) | **HCM**  (n=393) | | P value | **nHCM**  (n=292) | | **oHCM**  (n=101) | | P value |
| --- | --- | --- | --- | --- | --- | --- | --- | --- | --- |
| KCCQ-SB | 100 (83-100) | 83 (58-100) | | <.001 | 83 (58-100) | | 83 (58-100) | | 0.264 |
| KCCQ-TS | 100 (88-100) | 85 (65-100) | | <.001 | 85 (66-100) | | 83 (63-97) | | 0.191 |
| KCCQ-SE | 75 (50-100) | 75 (63-100) | | 0.993 | 75 (63-100) | | 75 (63-100) | | 0.429 |
| KCCQ-QoL | 100 (83-100) | 83 (58-100) | | <.001 | 83 (58-100) | | 71 (50-92) | | 0.051 |
| KCCQ-SL | 100 (100-100) | 85 (56-100) | | <.001 | 88 (56-100) | | 81 (63-94) | | 0.238 |
| KCCQ-CS | 98 (90-100) | 88 (67-98) | | <.001 | 89 (68-98) | | 83 (65-94) | | 0.036 |
| KCCQ-OS | 98 (90-100) | 85 (61-96) | | <.001 | 86 (62-97) | | 81 (60-93) | | 0.073 |
| EQ-5D-5L | 0.96 (0.85-1) | 0.88 (0.76-1) | | <.001 | 0.88 (0.77-1) | | 0.85 (0.73-1) | | 0.402 |
| EQ-VAS | 81 (70-90) | 77 (66-89) | | 0.006 | 80 (69-90) | | 73 (61-85) | | 0.190 |
|  |  | |  | | |  | |  | |
|  | **HCM NYHA I**  (n=235) | | **HCM NYHA II**  (n=134) | | | **HCM NYHA III**  (n=24) | | P value | |
| KCCQ-CS | 95 (85-100) | | 68 (55-85) | | | 60 (41-71) | | <.001 | |
| EQ-5D-5L | 0.91 (0.82-1.00) | | 0.78 (0.70-0.89) | | | 0.71 (0.65-0.81) | | <.001 | |
| EQ-VAS | 80 (70-90) | | 70 (60-81) | | | 63 (53-74) | | <.001 | |

**Supplemental Table S6** – Mean healthcare resource utilisation (HCRU) and mean costs per patient per year (PPPY) for all non-obstructive hypertrophic cardiomyopathy (nHCM) patients according to cost types. A total of 2000 replications were performed. Abbreviations: FC: friction costing; MRI: magnetic resonance imaging.

| **nHCM**  (n=288) | **HCRU** | **95% Bootstrapped CI HCRU** | **Costs PPPY**  (2023 Euro) | **95% Bootstrapped CI Costs** |
| --- | --- | --- | --- | --- |
| **Healthcare costs** |  |  | €7,173 | [€5,920, €8,611] |
| Primary healthcare |  |  | €861 | [€615, €1,165] |
| General Practitioner | 3.8 | [3.3, 4.3] | €125 | [€106, €146] |
| Practitioner assistant | 1.4 | [1.0, 1.8] | €28 | [€20, €37] |
| Social worker | 0.3 | [0.1, 0.7] | €38 | [€7, €92] |
| Physiotherapy | 8.3 | [5.4, 12.8] | €409 | [€228, €702] |
| Occupational therapy | 0.3 | [0.0, 0.6] | €6 | [€0, €15] |
| Dietitian | 0.6 | [0.4, 0.8] | €15 | [€8, €21] |
| Homeopathy | 0.2 | [0.1, 0.3] | €2 | [€0, €4] |
| Psychologist | 1.7 | [1.1, 2.4] | €192 | [€107, €290] |
| Company physician | 0.9 | [0.6, 1.1] | €29 | [€20, 40] |
| Homecare (hours) |  |  | €314 | [€65, €672] |
| Domestic support | 5.9 | [2.9, 10.5] | €215 | [€61, €444] |
| Self-care support | 1.1 | [0, 3.3] | €99 | [€0, €299] |
| Nursing support | 0 | [0, 0] | €0 | [€0, €0] |
| Emergency care |  |  | €284 | [€172, €413] |
| Emergency room | 0.6 | [0.4, 0.8] | €178 | [€112, €257] |
| Ambulance care | 0.2 | [0.1, 0.3] | €106 | [€53, €175] |
| Cardiologist care | 3.7 | [3.2, 4.1] | €459 | [€407, €512] |
| Other specialist care | 3.7 | [2.9 , 4.5] | €460 | [€362, €564] |
| Patient care |  |  | €3,617 | [€2,581, €4,840] |
| Cardiac echo | 3.2 | [2.9, 3.5] | €328 | [€294, €365] |
| Electrocardiogram | 4.0 | [3.6, 4.5] | €206 | [€179, €236] |
| MRI | 0.6 | [0.5, 0.8] | €138 | [€89, €192] |
| Blood testing | 3.3 | [2.7, 3.9] | €106 | [€83, €130] |
| Ergometry | 0.7 | [0.6, 0.9] | €100 | [€75, €128] |
| Holter monitoring | 1.1 | [1.0, 1.3] | €338 | [€277, €398] |
| Hospital procedures | 0.8 | [0.7, 1.0] | €1,879 | [€1,333, €2,599] |
| Hospital admissions (admissions per year) | 0.4 | [0.2, 0.5] | €1,178 | [€613, €1,978] |
| Residential home | 0 | [0, 0] | €0 | [€0, €0] |
| Rehabilitation centre | 0.4 | [0.1, 0.9] | €522 | [€37, €1,138] |
| Psychiatric centre | 0 | [0, 0] | €0 | [€0, €0] |
| Other centres | 0.1 | [0, 0.2] | €38 | [€0, €111] |
| Cardiac medications |  |  | €400 | [€339, €466] |
| Other medications |  |  | €813 | [€451, €1,283] |
| **Patient family costs** |  |  | €1,453 | [€787, €2,239] |
| Home care | 27.7 | [12.2, 47.8] | €541 | [€237, €932] |
| Self-care assistance | 10.0 | [2.9, 19.9] | €236 | [€26, €524] |
| Practical help | 23.2 | [12.0, 37.3] | €564 | [€232, €953] |
| Hospital travel costs |  |  | €111 | [€91, €135] |
| **Productivity losses** |  |  | €6,187 | [€4,793, €7,801] |
| Short-term sick leave |  |  | €634 | [€266, €1096] |
| Long-term sick leave, no FC |  |  | €1,381 | [€567, €2,326] |
| Long-term sick leave |  |  | €2,086 | [€1,113, €3,255] |
| Presenteeism |  |  | €1,011 | [€622, €1,475] |
| Disability costs |  |  | €1,074 | [€638, €1,560] |
| **Other sector costs** |  |  | €3,518 | [€2,231, €5,106] |
| **Mean total costs** |  |  | €18,330 | [€15,286, €21,593] |

**Supplemental Table S7** – Mean healthcare resource utilisation (HCRU) and mean costs per patient per year (PPPY) for all obstructive hypertrophic cardiomyopathy (oHCM) patients according to cost types. A total of 2000 replications were performed. Abbreviations: FC: friction costing; MRI: magnetic resonance imaging.

| **oHCM**  (n=101) | **HCRU** | **95% Bootstrapped CI HCRU** | **Costs PPPY**  (2023 Euro) | **95% Bootstrapped CI Costs** |
| --- | --- | --- | --- | --- |
| **Healthcare costs** |  |  | €7723 | [€5770, €10293] |
| Primary healthcare |  |  | €588 | [€418, €777] |
| General Practitioner | 4.2 | [3.1, 5.5] | €136 | [€100, €176] |
| Practitioner assistant | 1.9 | [1.0, 3.0] | €40 | [€22, €66] |
| Social worker | 0.6 | [0.1, 1.6] | €83 | [€5, €214] |
| Physiotherapy | 4.1 | [2.4, 6.3] | €167 | [€96, €256] |
| Occupational therapy | 0 | [0, 0] | €0 | [€0, €0] |
| Dietitian | 1.0 | [0.5, 1.6] | €25 | [€12, €41] |
| Homeopathy | 0.4 | [0, 1.0] | €11 | [€0, €30] |
| Psychologist | 0.9 | [0.3, 1.7] | €87 | [€28, €170] |
| Company physician | 1.1 | [0.6, 1.9] | €37 | [€18, €60] |
| Homecare (hours) |  |  | €265 | [€92,€476] |
| Domestic support | 7.2 | [2.2, 13.7] | €246 | [€75, €467] |
| Self-care support | 0 | [0, 0] | €0 | [€0, €0] |
| Nursing support | 0.2 | [0, 0.7] | €19 | [€0, €55] |
| Emergency care |  |  | €333 | [€150, €547] |
| Emergency room | 0.8 | [0.4, 1.3] | €203 | [€95, €339] |
| Ambulance care | 0.2 | [0.1, 0.4] | €130 | [€43, €239] |
| Cardiologist care | 4.9 | [4.0 , 5.9] | €614 | [€503, €740] |
| Other specialist care | 3.8 | [2.8 , 5.0] | €475 | [€345, €617] |
| Patient care |  |  | €3503 | [€2305, €5065] |
| Cardiac echo | 4.7 | [3.8, 5.7] | €501 | [€402, €614] |
| Electrocardiogram | 5.8 | [4.7, 7.0] | €291 | [€238, €354] |
| MRI | 1.0 | [0.7, 1.5] | €286 | [€187, €406] |
| Blood testing | 4.8 | [3.3, 6.6] | €155 | [€107, €213] |
| Ergometry | 1.3 | [0.9, 1.9] | €203 | [€137, €281] |
| Holter monitoring | 1.6 | [1.1, 2.2] | €456 | [€323, €613] |
| Hospital procedures | 1.1 | [0.6, 1.6] | €2287 | [€1795, €2915] |
| Hospital admissions (admissions per year) | 0.4 | [0.2, 0.8] | €930 | [€159, €2065] |
| Residential home | 0 | [0, 0] | €0 | [€0, €0] |
| Rehabilitation centre | 0.3 | [0, 0.9] | €285 | [€0, €803] |
| Psychiatric centre | 0 | [0, 0] | €0 | [€0, €0] |
| Other centres | 0 | [0, 0] | €0 | [€0, €0] |
| Cardiac medications |  |  | €303 | [€226, €392] |
| Other medications |  |  | €1657 | [€493, €3703] |
| **Patient family costs** |  |  | €1262 | [€581, €2065] |
| Home care | 26.1 | [5.3, 54.1] | €509 | [€104, €1056] |
| Self-care assistance | 8.0 | [0.3, 20.0] | €157 | [€6, €389] |
| Practical help | 21.0 | [3.0, 47.7] | €411 | [€59, €931] |
| Hospital travel costs |  |  | €185 | [€129, €252] |
| **Productivity losses** |  |  | €8,352 | [€5,018, €12,507] |
| Short-term sick leave |  |  | €2211 | [€916, €3746] |
| Long-term sick leave, no FC |  |  | €1,546 | [€98, €3,341] |
| Long-term sick leave |  |  | €931 | [€0, €2344] |
| Presenteeism |  |  | €2899 | [€969, €5485] |
| Disability costs |  |  | €766 | [€163, €1470] |
| **Other sector costs** |  |  | €3698 | [€1454, €6400] |
| **Mean total costs** |  |  | €21,036 | [€15,602, €27,474] |

**Supplemental Table S8** – Mean healthcare resource utilisation (HCRU) and mean costs per patient per year (PPPY) for all genotype-positive phenotype-negative (G+/P-) subjects according to cost types. A total of 2000 replications were performed. ✝: costs for these investigations were not extrapolated in G+/P- subjects. Abbreviations: FC: friction costing; MRI: magnetic resonance imaging.

| **G+/P-**  (n=76) | **HCRU** | **95% Bootstrapped CI HCRU** | **Costs PPPY**  (2023 Euro) | **95% Bootstrapped CI Costs** |
| --- | --- | --- | --- | --- |
| **Healthcare costs** |  |  | €1873 | [€1147, €2732] |
| Primary healthcare |  |  | €692 | [€399, €1077] |
| General Practitioner | 2.6 | [1.9, 3.4] | €83 | [€61, €108] |
| Practitioner assistant | 1.0 | [0.4, 1.9] | €21 | [€8, €41] |
| Social worker | 0.1 | [0, 0.2] | €7 | [€0, €21] |
| Physiotherapy | 7.6 | [3.6, 12.5] | €308 | [€147, €506] |
| Occupational therapy | 0.8 | [0, 2.1] | €21 | [€0, €52] |
| Dietitian | 0.1 | [0, 0.2] | €1 | [€0, €4] |
| Homeopathy | 0.5 | [0.1, 1] | €15 | [€2, €32] |
| Psychologist | 2.2 | [0.6, 4.4] | €226 | [€59, €447] |
| Company physician | 0.3 | [0.1, 0.6] | €10 | [€2, €20] |
| Homecare (hours) |  |  | €149 | [€0, €396] |
| Domestic support | 2.6 | [0, 6.6] | €90 | [€0, €226] |
| Self-care support | 0.4 | [0, 1.3] | €25 | [€0, €75] |
| Nursing support | 0.4 | [0, 1.3] | €33 | [€0, €98] |
| Emergency care |  |  | €28 | [€0, €70] |
| Emergency room | 0.1 | [0, 0.3] | €28 | [€0, €70] |
| Ambulance care | 0 | [0, 0] | €0 | [€0, €0] |
| Cardiologist care✝ | 0.3 | [0.2 , 0.6] | €44 | [€25, €69] |
| Other specialist care | 1.9 | [1.0 , 3.0] | €242 | [€124, €374] |
| Patient care |  |  | €566 | [€234, €985] |
| Cardiac echo✝ | 0.4 | [0.3, 0.6] | €45 | [€32, €59] |
| Electrocardiogram✝ | 0.4 | [0.3, 0.5] | €19 | [€13, €26] |
| MRI✝ | 0.1 | [0.1, 0.2] | €33 | [€15, €55] |
| Blood testing✝ | 0.3 | [0.2, 0.5] | €11 | [€6, €16] |
| Ergometry✝ | 0.1 | [0.0, 0.1] | €10 | [€2, €22] |
| Holter monitoring✝ | 0.1 | [0.0, 0.1] | €18 | [€4, €33] |
| Hospital procedures | 0.5 | [0.2, 0.8] | €849 | [€520, €1254] |
| Hospital admissions (admissions per year) | 0.1 | [0, 0.2] | €109 | [€0, €317] |
| Residential home | 0 | [0, 0] | €0 | [€0, €0] |
| Rehabilitation centre | 0 | [0, 0] | €0 | [€0, €0] |
| Psychiatric centre | 0 | [0, 0] | €0 | [€0, €0] |
| Other centres | 0.1 | [0, 0.2] | €18 | [€0, €54] |
| Cardiac medications |  |  | €21 | [€10, €35] |
| Other medications |  |  | €312 | [€29, €806] |
| **Patient family costs** |  |  | €273 | [€76, €561] |
| Home care | 5.7 | [0.6, 12.6] | €110 | [€12, €247] |
| Self-care assistance | 2.9 | [0, 8.8] | €57 | [€0, €173] |
| Practical help | 2.3 | [0, 6.7] | €45 | [€0, €131] |
| Hospital travel costs |  |  | €60 | [€27, €106] |
| **Productivity losses** |  |  | €3,282 | [€1,382, €5,538] |
| Short-term sick leave |  |  | €352 | [€0, €935] |
| Long-term sick leave, no FC |  |  | €1,383 | [€0, €3,274] |
| Long-term sick leave |  |  | €606 | [€0, €1811] |
| Presenteeism |  |  | €825 | [€330, €1458] |
| Disability costs |  |  | €441 | [€0, €1081] |
| **Other sector costs** |  |  | €1,957 | [€187, €4,754] |
| **Mean total costs** |  |  | €7,385 | [€3,913, €11,870] |

**Supplemental Table S9** – Bootstrapped primary cost (per patient per year, PPPY) groups for all hypertrophic cardiomyopathy (HCM) patients grouped by NYHA (New York Health Association) class.

| **Costs PPPY**  Bootstrapped  PPPY [95%CI] | | **HCM NYHA I**  (n=233) | **HCM NYHA II**  (n=133) | **HCM NYHA III**  (n=23) | |
| --- | --- | --- | --- | --- | --- |
| Healthcare costs | €6,361  [€5,049, €7,780] | | €8,645  [€6,859, €10,783] | | €8,954  [€4,089, €16,923] |
| Patient family costs | €834  [€404, €1,326] | | €2,229  [€955, €3,775] | | €2,600  [€232, €5,723] |
| Productivity losses | €5,157  [€3,612; €6,778] | | €7,587  [€5,320, €10,150] | | €8,930  [€2,050, €19,661] |
| Other sector costs | €1,659  [€777, €2,758] | | €5,619  [€3,290, €8,327] | | €11,436  [€2,868, €22,634] |
| Mean total costs | €14,012  [€10,995, €17,225] | | €24,080  [€19,447, €29,153] | | €31,920  [€16,790, €47,877] |

**Supplemental Table S10** – Mean (with Q1-Q3 interquartile ranges) primary cost (per patient per year, PPPY) groups for all genotype-positive phenotype-negative (G+/P-) subjects, and hypertrophic cardiomyopathy (HCM) patients grouped by NYHA (New York Health Association) class.

| **Costs PPPY**  Mean [IQR] | | **G+/P-**  (n=76) | | **HCM NYHA I**  (n=233) | | **HCM NYHA II**  (n=133) | **HCM NYHA III**  (n=23) |
| --- | --- | --- | --- | --- | --- | --- | --- |
| Healthcare costs | €2563  [€172, 3163] | | €6367  [€1518, €5910] | | €8664  [€2169, €11305] | | €8938  [€2308, €8183] |
| Patient family costs | €272  [€0, €84] | | €838  [€0, €156] | | €2210  [€3, €426] | | €2548  [€15, €328] |
| Productivity losses | €3616  [€0, €1149] | | €6050  [€0, €2002] | | €7560  [€0, €13028] | | €8963  [€0, €15843] |
| Other sector costs | €1929  [€0, €0] | | €1666  [€0, €0] | | €5575  [€0, €2156] | | €11295  [€0, €10147] |
| Mean total costs | €8381  [€217, €5478] | | €14921  [€1953, €14646] | | €24009  [€3461, €36595] | | €31744  [€4997, €48776] |

| **Costs PPPY**  Mean [IQR] | **G+/P-**  (n=76) | **HCM**  (n=389) | **P value** |
| --- | --- | --- | --- |
| Healthcare costs | €2563  [€172, 3163] | €7305  [€1903, €7627] | <.001 |
| Patient family costs | €272  [€0, €84] | €1408  [€0, €259] | <.001 |
| Productivity losses | €3616  [€0, €1149] | €6739  [€0, €4504] | 0.022 |
| Other sector costs | €1929  [€0, €0] | €3572  [€0, €0] | 0.283 |
| Mean total costs | €8381  [€217, €5478] | €19023  [€2346, €21886] | <.001 |

**Supplemental Table S11** – Sensitivity and scenario analyses compared to base case results. Abbreviations: PPPY: per patient per year; CI: confidence interval; G+/P-: genotype positive, phenotype negative; HCM: hypertrophic cardiomyopathy.

|  | **Sub-analyses** | | **Base case** | |  |
| --- | --- | --- | --- | --- | --- |
|  | Bootstrapped mean costs (PPPY) | 95% CI intervals | Bootstrapped mean costs (PPPY) | 95% CI intervals |  |
| SA1 | Healthcare perspective | | Societal perspective | |  |
|  | G+/P- (n =76) | HCM patients (n = 389) | G+/P- (n = 76) | HCM patients (n = 389) |  |
|  | €1,873 [€1,147, €2,732] | €7,330 [€6,334, €8,498] | €7,385 [€3,913, €11,870] | €19,035 [€16,299, €21,974] |  |
| SA2 | Patient care costs HCM without extrapolation of cardiovascular examinations | | Patient care costs HCM with extrapolation of cardiovascular examinations | |  |
|  | HCM patients (n = 389) | | HCM patients (n = 389) | |  |
|  | €2,989 [€1973, €4,186] | | €3,581 [€2,763, €4,525] | |  |
| SA3 | Non-cardiac medication costs without cut-off point for expensive medications | | Non-cardiac medication costs with cut-off point for expensive medications | |  |
|  | G+/P- (n =76) | HCM patients (n = 389) | G+/P- (n =76) | HCM patients (n = 389) |  |
|  | €312 [€29, €806] | €1,078 [€579, €1697] | €312 [€29, €806] | €921 [€505, €1,481] |  |
| SA4 | Productivity losses calculated with  human capital approach | | Productivity losses calculated with  friction cost approach (stop after 19 weeks) | |  |
|  | G+/P- (n =76) | HCM patients (n = 389) | G+/P- (n =76) | HCM patients (n = 389) |  |
|  | €5,969  [€2199, €10,892] | €11,605 [€9,034, €14,338] | €3,282  [€1,382, €5,538] | €6,724  [€5,365, €8,284] |  |

**Supplemental Figure S1** – Correlation plots between the questionnaires and the HCM SCD Risk score for all HCM patients without prior septal reduction therapy and complete questionnaire data. Abbreviations: EQ-5D-5L: EuroQoL 5-domain, 5-level; EQ-VAS: EuroQol Visual analogue scale; HCM: hypertrophic cardiomyopathy; KCCQ: Kansas City Cardiomyopathy Questionnaire; SCD: sudden cardiac risk.


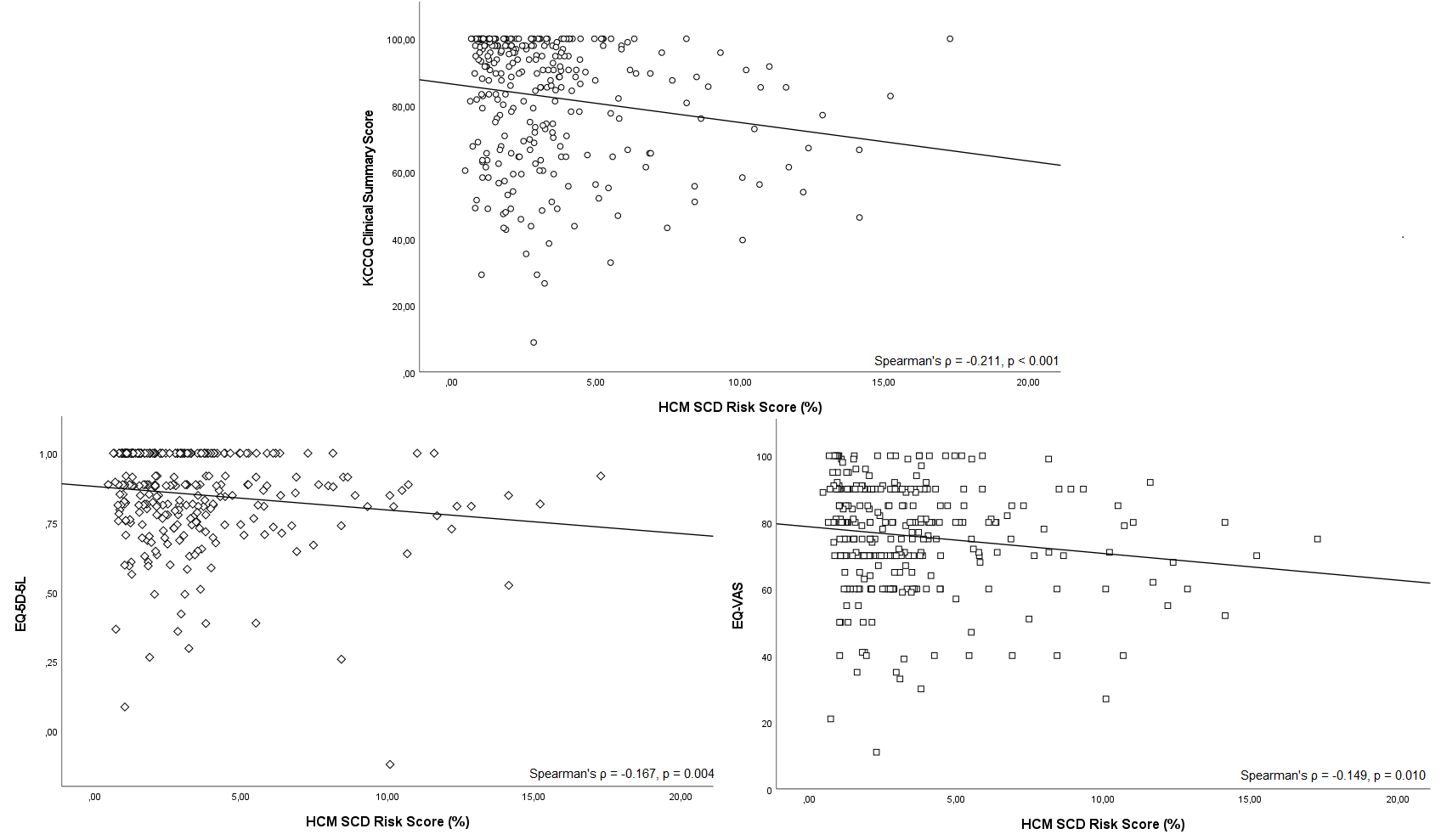


**Supplemental Figure S2** – Illustration of the subcategories of all costs of both main subject groups stratified by age. A: Healthcare costs; B: patient and family costs; C: productivity costs; D: other sector
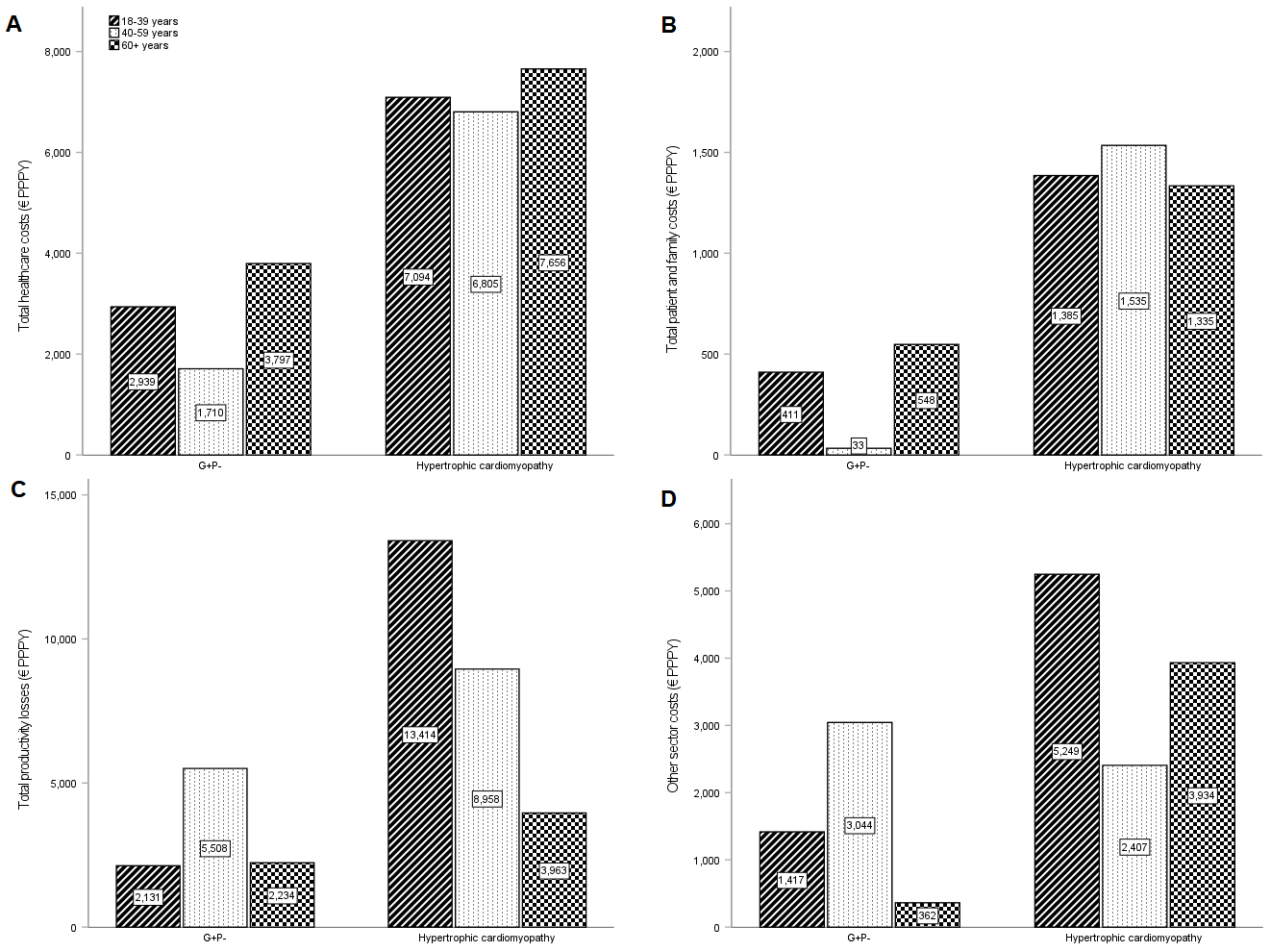
costs. Abbreviation: G+/P-: genotype-positive, phenotype-negative subject; PPPY: per patient per year.

**Supplemental Figures S3-S4** – Tornado diagram for sensitivity analyses. Abbreviations**:** SA1 = healthcare perspective; SA2 = patient care costs for HCM without extrapolation of cardiovascular examinations; SA3 = non-cardiac medication costs without cut-off point for expensive medications; SA4 = productivity losses calculated with human capital approach.

* Base case for HCM was €19,035

* Base case for G+/P- was €7,385
