# Supplementary figures and images for: Loss of quality of life and increased societal costs in patients with hypertrophic cardiomyopathy: the AFFECT-HCM study
Source: Eur Heart J Qual Care Clin Outcomes. 2024 Nov 8;11(2):174–85. doi: 10.1093/ehjqcco/qcae092 (PMC11879321; doi:10.1093/ehjqcco/qcae092)

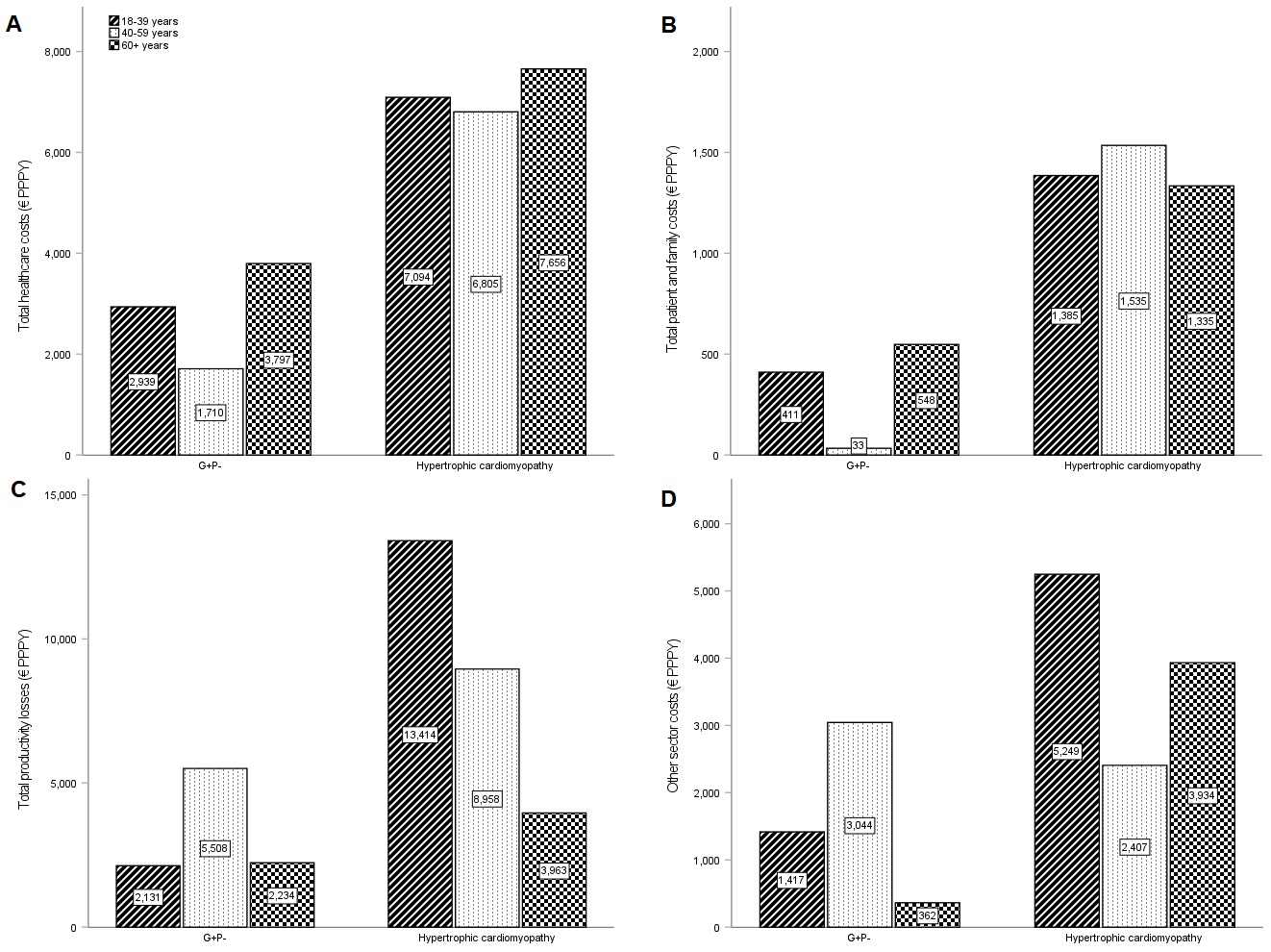

Supplement: qcae092_Supplemental_Files [file qcae092_supplemental_files.zip › Supplemental Figure S1.jpg]
